# Growth promotion and stress tolerance of soybean plants driven by seed treatment with synthetic bacterial community of soybean-associated beneficial bacteria

**DOI:** 10.3389/fpls.2025.1729743

**Published:** 2026-01-07

**Authors:** Rosalie B. Calderon, Sandeep Gouli, Inderjit Barphagha, Jong Hyun Ham

**Affiliations:** Department of Plant Pathology and Crop Physiology, Louisiana State University Agricultural Center, Baton Rouge, LA, United States

**Keywords:** soybean microbiome, synthetic community, plant-microbiome interactions, seed treatment, enhanced disease resistance, enhanced drought tolerance, enhanced waterlogging tolerance

## Abstract

**Introduction:**

Beneficial microbes provide a sustainable approach to improving crop production and reducing risks from intensive farming. Microbial consortia, complementary traits, often outperform single strains in promoting plant health. This study aimed to develop an effective biological strategy to enhance soybean growth and health using beneficial bacterial consortia.

**Methods:**

Bacteria were isolated from the root endosphere and rhizosphere of field-grown soybean plants and screened for traits such as nutrient solubilization, hormone production, and pathogen suppression. Seven synthetic bacterial communities (SBCs), each comprising 5 to 20 SABB strains, were constructed to evaluate their potential in promoting soybean growth and health. Impact of SBC seed treatments on the structure of soybean microbiota was also investigated.

**Results:**

Two SBC sets, Set2 and Setm4, demonstrated superior performances in enhancing plant growth and resistance to the fungal pathogen *Rhizoctonia solani* when applied via seed treatment. Notably, seed treatment with Set2 or Setm4 also improved soybean resilience to abiotic stresses, including drought and waterlogging. Profiling of the root endosphere and rhizosphere microbiota revealed that SBC application through seed treatment significantly altered the composition of soybean-associated microbial community, including the enrichment of key symbiotic taxa, such as *Bradyrhizobium elkanii*, and increased microbial network complexity.

**Discussion:**

The beneficial effects of SBC through seed treatment are closely related to microbiome restructuring in soybean roots. This study provides valuable insights into the development of innovative and sustainable crop management strategies, highlighting the potential of SBC-based seed treatments to enhance growth and stress resilience in soybeans and other major crops.

## 1 Introduction

Exploiting the potential of beneficial microbes stands out as a promising strategy to enhance the sustainability of crop production, mitigating risks associated with intensified agricultural practices (Busby et al., 2017; Chouhan et al., 2021; Singh et al., 2018, 2020). While the exploration of beneficial microbes for crop growth and protection has traditionally focused on the specific biological activity of single microorganisms (De Souza et al., 2015; Lugtenberg and Kamilova, 2009; Qiu et al., 2019; Sébastien et al., 2015; Ray et al., 2020), recent studies have demonstrated that the collective activities of multiple beneficial microbes can outperform individual members in enhancing crop productivity (Ma et al., 2024; You et al., 2025; Xu et al., 2025; Chouhan et al., 2021; Trivedi et al., 2020). Unlike individual strains, microbial consortia combine complementary traits such as nutrient solubilization, phytohormone production, nitrogen fixation, and antifungal metabolite synthesis, leading to synergistic effects and broader functional coverage (Khan et al., 2022; Thingujam et al., 2025). It was reported that commonly used genera, including *Bacillus, Pseudomonas, Rhizobium*, and *Enterobacter*, collectively enhance nutrient uptake, induce systemic resistance, and improve root colonization, where *Rhizobium* occupies nodules for nitrogen fixation, while *Bacillus* and *Pseudomonas* dominate the rhizosphere and suppress pathogens (Santoyo et al., 2021). Other studies also demonstrated that microbial consortia significantly improved plant performance by promoting root and shoot biomass, enhancing photosynthetic efficiency, accelerating nutrient cycling, and increasing productivity, indicating their potential as biofertilizers in conventional agricultural systems (Lobo et al., 2019; Moretti et al., 2024; Parveen and Ghosh, 2025).

Microbial consortia have also shown strong efficacy in mitigating biotic stresses. Combined application of *Bacillus subtilis* and *Pseudomonas fluorescens* suppressed the fungal pathogen *Rhizoctonia solani* in tomato, while co-inoculation of *Rhizobium* and *Bacillus* reduced root rot in beans and improved yield (De Jensen et al., 2002; Mezeal, 2014). Microbial consortia can also enhance abiotic stress tolerance by promoting osmotic adjustment, regulating antioxidant enzymes (such as catalase and peroxidases), and reducing oxidative damage, particularly under drought, salinity, and heat stress. For instance, drought-resilient consortia composed of *Enterobacter*, *Bacillus cereus*, and *Bacillus megaterium* improved wheat growth by enhancing root development, chlorophyll retention, and membrane stability (Shankar and Prasad, 2023). Similarly, it was reported that saline-stress mitigation by co-cultured *Pseudomonas koreensis* and *Microbacterium hydrothermale* was attributed to enhanced ACC deaminase activity, suppressed ethylene accumulation, and improved hormonal regulation (Choudhury et al., 2021). In spinach and wheat, *Bacillus*-based consortia also enhanced drought and salt tolerance by stabilizing K^+^/Na^+^ homeostasis, promoting seedling establishment and improving chlorophyll content (Khan et al., 2022; Petrillo et al., 2022). These synergistic outcomes are largely driven by shared production of lipopeptides, metabolites, siderophores, and phytohormones, resulting in broad-spectrum pathogen inhibition and optimized growth and defense regulation (Timofeeva et al., 2023).

Microbiome studies on soybean (*Glycine max* L.), one of the most important crops worldwide, have predominantly focused on taxonomical characterization in various plant compartments, such as seed, phyllosphere, root nodules, and rhizosphere (Yang et al., 2024; Rascovan et al., 2016; Díaz-Cruz and Cassone, 2022), or at different growth stages (Longley et al., 2020; Cheng et al., 2022). Other research areas include temporal and spatial dynamics of a microbial community influenced by crop management practices (Díaz-Cruz and Cassone, 2022; Bell-Dereske et al., 2023; Moroenyane et al., 2021; Longley et al., 2020; Calderon et al., 2021). While some studies have characterized natural or synthetic microbial communities that suppress diseases or enhance nutrient uptake (Mendes et al., 2011; Hussain et al., 2024; Wang et al., 2021), our understanding of the soybean microbiome structure underlying their beneficial impacts on soybean growth and health is still limited. Nevertheless, recent advances in the study of plant-associated microorganisms have shifted towards a systems-level understanding of the collective functionality of microbiome, aiming to improve host health and vigor (Compant et al., 2025; Dai et al., 2025; Trivedi et al., 2021; Nyholm et al., 2020; Xu et al., 2021).

In this study, we investigated the beneficial effects of bacterial consortia comprised of assorted soybean-associated beneficial bacteria (SABB) on soybean growth and resilience to biotic and abiotic stresses, especially through seed treatment, and explored the dynamics of soybean microbiome profiles associated with their beneficial activities.

## 2 Materials and methods

### 2.1 Isolation, screening, and identification of soybean-associated beneficial bacteria

The soybean roots and rhizospheric soil samples were collected from robust and visibly healthy plants in soybean fields located at the Doyle Chambers Central Research Station (Baton Rouge, LA), the Red River Research Station (Bossier City, LA), and the H. Rouge Caffey Rice Research Station (Rayne, LA) in Louisiana. Samples were transported in a cooler and stored at temperatures ranging from 4°C to 15°C. Soybean-associated bacteria (SAB) were isolated within 48h after sample collection, following the protocol described by Sasser et al (Sasser et al., 1990).

Antagonistic capabilities of the bacterial isolates were assessed through dual-plate confrontation assays, as previously described by Shrestha et al (Shrestha et al., 2016). Additionally, SAB were screened for other beneficial activities for plant growth, including nitrogen fixation, indole-3-acetic acid (IAA) production, phosphate solubilization, siderophore production, and starch hydrolysis, using previously described methods (Jensen, 1942) (Pikovskaya, 1948; Gordon and Weber, 1951; Alexander and Zuberer, 1991; Shruti et al., 2013) (see the ‘**Supplementary Information**’ for detailed methodology).

Selected bacterial isolates exhibiting robust beneficial activities were identified based on their 16S rRNA gene sequences. DNA extraction was performed using Qiagen DNA kit (Qiagen, Germantown, MD, USA), and the 16S rRNA gene was amplified using the forward primer fD1 (5’-AGAGTTTGATCCTGGCTCAG-3’) and the reverse primer rP2 (5’-ACGGCTACCTTGTTACGACTT-3’) (Weisburg et al., 1991). Sequencing of PCR products was conducted by MACROGEN, Inc. (Seoul, Korea), and the DNA sequencing data were analyzed for homology to identify corresponding bacterial taxa, using the BLAST program of the NCBI website (http://blast.ncbi.nlm.nih.gov/Blast.cgi). The phylogenetic tree was constructed using Randomized Axelerated Maximum Likelihood (RAxML), following the methodology outlined by Stamatakis (2014).

### 2.2 *In vitro* co-culture compatibility assay, formulation, and evaluation of bacterial consortia

Compatibility of bacterial strains was determined utilizing the cross-streak test as described by Santiago et al. (2017), where compatibility was indicated by normal colony development of both strains at the junction point, while the presence of inhibition zone or lysis at the junction point was considered as incompatibility.

Synthetic bacterial communities (SBCs) were prepared by combining equal volumes of compatible strains adjusted to OD_600_ = 1.0 (> 1 X 10^9^ CFU/ml). Subsequently, the formulated SBCs were systemically evaluated under laboratory, greenhouse, and field conditions. Statistical analyses were performed using JMP Pro Statistics, version 15.1.0 (SAS Institute, Cary, NC).

### 2.3 Evaluation of the soybean growth-promoting and defense-enhancing activities of SAB and SBCs

The direct growth-promoting activity of individual SAB was assessed using plastic pouches in the laboratory (**Supplementary Figure S1**). Initially, seeds were sterilized with 30% hydrogen peroxide for 5 min, after which the seeds were washed extensively with sterile water (Copley et al., 2017). Soybean seeds were then inoculated with bacterial suspensions (from 24h-old bacterial cultures) in 10 mM MgCl_2_ amended with 2% carboxymethyl cellulose (CMC) in 250-ml flasks and incubated on a shaker at 180 rpm for 30 min at 30 ± 2°C, while the control seeds were treated with an equal amount of the 10 mM MgCl_2_ and 2% CMC solution. After incubation, the treated seeds were placed in a Petri dish and air dried under a laminar flow overnight (12-16 h). Sterile plastic pouches containing moistened paper towels were seeded with untreated seeds as a control, alongside seeds treated with individual SAB isolates or SBCs adjusted to OD_600_ = 1.0 (> 1 X 10^9^ CFU/ml). These pouches were then placed on the plant-growing shelves set in the laboratory (25°C, 14 h light/day) and watered with sterile water.

Seedling rot assays were performed using sterile pouches and sterile paper towels in a growth chamber condition set as 25°C/23°C (day/night), 14 h light period, and 400- 600 µmol light intensity. *Rhizoctonia solani* AG1 was cultured on potato dextrose agar plates (PDA) at 30°C in the dark in an incubator for 3 to 5 days. A 5 mm-diameter mycelial plug of *R. solani* was placed at the root-shoot junction of the 10-day-old seedling and wrapped with the aluminum foil to maintain humidity. Sterile 5-mm-diameter PDA plugs were used for mock inoculation. Symptom development caused by the fungal pathogen *R. solani* was assessed using a disease severity rating scale ranging from 1 to 5, in which 1 indicates no root rot; 2 represents 1 to 33% of roots with visible lesions or root rot; 3 corresponds to approximately 33 to 50% of the roots rotted or damaged; 4 indicates approximately 51 to 75% of the roots affected; and 5 denotes preemergence damping-off with few, if any, roots present (Dorrance et al., 2003). The performances of the SAB isolates and SBCs in the pouch assay were validated through greenhouse experiments.

In the greenhouse condition, two sets of experiments were conducted for assessing resistance to *R. solani* and growth promotion with 10-day-old and 28-day-old plants, respectively. For the disease assay with *R. solani*, soybean seeds treated with either individual SABB or mixtures (SBCs) or the untreated seeds as control were sown in 10 cm x 10 cm square pots filled with the river silt soil provided by the Plant Material Center of LSU AgCenter. Prior to each trial, the soil was sterilized three consecutive days at 121 °C for 90 min. Disease rating was conducted 7 days after inoculation of the seedlings. For assessing growth promotion, treated or untreated seeds were directly sown into 28 cm diameter pots filled with field soil. The treatments were arranged in a Randomized Complete Block Design (RCBD) with four replications. After 4 weeks, plant growth and nodule formation were assessed, using a ruler and a WinRHIZO root scanner (Regent Instruments Inc., Canada), respectively.

Field trial was conducted at the field plot of the Doyle Chambers Central Research Station, Baton Rouge, Louisiana (30°21’38.8”N 91°10’14.6”W) to evaluate the effect of Set2 and Setm4 SBCs in comparison with untreated treatments and commercial seed-treating fungicide (Apron Maxx RTA, Syngenta). Due to the large volume required in the field, seeds were not surface sterilized prior to the seed treatment of the SBCs. Each treatment was planted in a regular plot dimension of 3.05 m width by 7.62 m length in raised beds. Five replications were imposed for each treatment following RCBD. The field plots were maintained following regular cultural management but without fertilization or pesticide application. One-way ANOVA followed by Tukey’s HSD test was used for statistical analysis of data for plant growth, disease severity, and yield.

### 2.4 Assays for soybean tolerance to drought and waterlogging

The effectiveness of bacterial consortia (Set2 and Setm4) in enhancing soybean tolerance to abiotic stresses was evaluated under greenhouse conditions. Seeds treated with Set2 or Setm4, along with untreated controls (UTC), were sown in a 1:1 soil-to-sand mixture (pH 6.8) in 19-cm circular pots, with six seeds per pot and 30 seeds per treatment arranged in a completely randomized design (CRD). Following emergence, 15 and 12 healthy seedlings per treatment were selected for drought and flooding stress applications, respectively. Flooding stress was imposed for 0, 4, or 7 days by placing pots in trays containing stagnant water to maintain soil moisture at 55-65%, whereas drought stress was induced 3–4 days after emergence by withholding water until soil moisture decreased to 10-15% as measured with a soil moisture meter, and was maintained for 4 and 7 days. At the end of each stress period, seedlings were carefully uprooted, roots were washed to remove adhering soil, and root and shoot lengths were measured with a calibrated ruler, while fresh weight was recorded immediately after blotting excess moisture. Dry weight was determined by oven-drying seedlings at 55 ^0^C for 45 h. Data on root length, shoot length, fresh weight, and dry weight were analyzed using one-way ANOVA followed by Tukey’s HSD test to identify significant differences among treatments, thereby evaluating the role of Set2 and Setm4 in mitigating the adverse effects of flooding and drought stress in soybean.

### 2.5 Mesocosm study to characterize the soybean microbial community associated with growth promotion

A mesocosm experiment was conducted to characterize the soybean-associated microbial community using field-collected soil under greenhouse conditions. The experiment utilized the field soil obtained from the Doyle Chambers Central Research Station in Baton Rouge, Louisiana, which showed slightly neutral pH and contained moderate organic matters (**Supplementary Table S1**).

Methodology for collecting samples of the root endosphere and the rhizosphere were conducted following Edwards et al. (2015). Soil samples from the rhizosphere and the root tissue samples for the root endosphere were processed for DNA extraction using the Qiagen PowerSoil^®^ DNA Isolation Kit (Qiagen, USA). The quantity and quality of the extracted DNA samples were assessed using NanoDrop 1000 (Thermo Scientific, Wilmington, DE, USA). The final DNA samples were sent to either the Genomics Research Laboratory of the Biocomplexity Institute of Virginia Tech (Blacksburg, VA, USA) or the Microbiome Services of the University of Minnesota Genomics Center for sequencing. Sequencing followed the 16S Illumina Amplicon Protocol from the Earth Microbiome Project (https://earthmicrobiome.org/protocols-and-standards/16s/), with amplification of the V4 -V5 region of the 16S rRNA gene using the 515F/926R primer pair (Parada et al., 2016; Quince et al., 2011) and sequencing on the MiSeq platform (Illumina, Inc., San Diego, CA).

Analysis of microbial community was performed using the Quantitative Insights into Microbial Ecology (QIIME 2) pipeline (Estaki et al., 2020). The ‘q2‐diversity’ plugin was utilized for alpha diversity and beta diversity metric calculations after rarefaction of samples at an even sampling depth. The rarefaction curves of the microbiome plateau, indicating a sufficient proportion of diversity represented in the sampling, are presented in **Supplementary Figure S2**. Statistically significant differences in microbial community structure were assessed using permutational multivariate analysis of variance (PERMANOVA) based on multivariate distance matrices with 999 permutations. Because PERMANOVA can be sensitive to differences in within-group dispersion, we also evaluated the homogeneity of multivariate dispersions using the permutational analysis of multivariate dispersions (PERMDISP) test, which formally assesses whether the average distance of samples to their respective group centroid is equal across groups. Core microbiome analysis was conducted using the core function in the R package microbiome (Lahti and Shetty, 2017). Datasets from QIIME output were further visualized using microbiomeAnalyst (Chong et al., 2020).

### 2.6 DNA sequence analysis to characterize differential abundance patterns of individual SABB strains within the SBCs (Set2 and Setm4)

Monitoring individual SABB strains of Set2 and Setm4 in the soybean microbiota was conducted based on sequence-read similarities. Prior to seed treatment, a portion of each SBC, prepared with pure-cultured SABB, underwent the same procedure to obtain the amplicon sequence profile corresponding to the same V4-V5 region. This profile served as a reference sequence, indicating the original composition of the SBC and facilitating the identification of its SABB components in the microbiome sequences. The ‘species’ instead of ‘genus’ taxonomic rank was chosen to identify the SABB strains of the SBC treated based on the sequence read, with the highest sequence similarity in the microbiome.

### 2.7 Statistical analysis

Differential abundance of bacterial species was determined using DESeq2 package in R (Love et al., 2014). Random Forest (RF) in R package was used to find the most significant taxa in the microbiome (Liaw and Wiener, 2002).

The co-occurrence network analysis was performed using the “igraph” package in R (Csardi and Nepusz, 2006). Correlation between two amplicon sequence variants (ASVs) was considered statistically significant at > 0.6 of Spearman’s correlation coefficient (r_s_) and < 0.01 of the *p* value (Barberán et al., 2012; Junker and Screiber, 2008; Ju et al., 2014). Benjamini-Hochberg standard false discovery rate correction method was performed using multiple correction to adjust the *p* values and reduce the chance of obtaining false-positive results (Benjamini and Hochberg, 1995). Topological properties and statistical analyses were calculated with R using vegan (Ihaka and Gentleman, 1996) and Hmisc (Harrell, 2008) packages. The network was visualized using Gephi platform (http://gephi.github.io/) (Bastian et al., 2009). Bacterial co-occurrence interaction patterns were determined using a python script previously developed by Ju and Zhang (2015).

## 3 Results

### 3.1 Selection of soybean-associated beneficial bacteria

**Figure 1:**
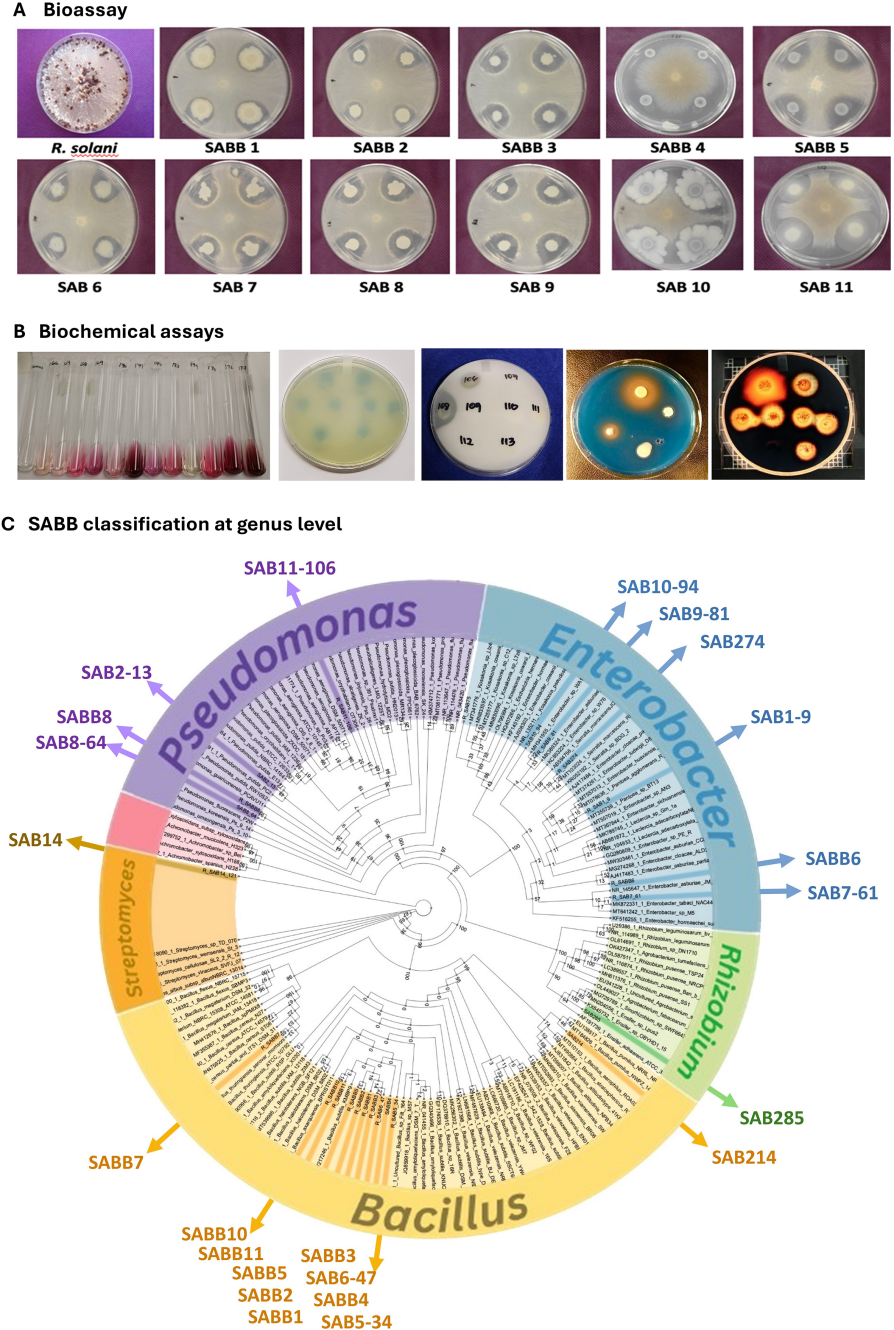
Beneficial activities and taxonomic groups of soybean-associated beneficial bacteria (SABB) identified in this study. **(A)** Antifungal activities against *Rhizoctonia solani*, **(B)** Beneficial activities for plant growth: (from left to right) indole acetic acid (IAA) production, nitrogen fixation, phosphate solubilization, siderophore production, and starch hydrolysis. **(C)** Classification of SABB at the genus level. The phylogenetic tree was constructed based on 16S rRNA genes using Randomized Axelerated Maximum Livelihood (RAxML). Scientific names embedded within the panel **(C)** are not italicized because their font style is fixed in the output images.

A total of 1,740 bacterial isolates were obtained from the root endosphere and the rhizosphere of soybean plants. Of these ‘soybean-associated bacteria (SAB)’, 345 isolates were screened as ‘soybean-associated beneficial bacteria (SABB)’ based on their antifungal and other beneficial activities (**Figures 1A, B**). This culture collection included 141 isolates that inhibited *Rhizoctonia solani* in culture (41%), 104 isolates that promoted seedling growth (30%), 279 isolates that could fix nitrogen (81%), 266 isolates that produced IAA (77%), 41 isolates that produced siderophore (12%), 41 isolates that produced amylase (12%), and 14 isolates that solubilized phosphate (4%). Some of these isolates exhibited multiple beneficial traits. Specifically, 49 isolates (14.2%) exhibited nitrogen fixation, IAA production, and siderophore synthesis. A broader range of activities was observed in 43 isolates (12.46%), which combined nitrogen fixation, IAA production, amylase activity, siderophore synthesis, and antifungal properties. Additionally, 41 isolates (11.88%) exhibited nitrogen fixation and IAA production. Furthermore, 26 isolates showed nitrogen fixation, IAA production, and siderophore synthesis along with amylase activity, whereas 25 isolates displayed the same traits but lacked amylase activity. Notably, strains belonging to the genera *Bacillus, Pseudomonas, Streptomyces, Enterobacter, Kosakonia, Leclercia, Ensifer, Rhizobium*, and *Achromobacter* demonstrated multiple beneficial traits. These strains were preferentially included in SBC construction to maximize synergistic effects among different plant growth-promoting traits (**Figure 1C**). Nevertheless, none of these SABB exhibited consistent growth-promoting activity alone through seed treatment in greenhouse or field conditions. Thus, we designed a series of SABB mixtures to test if multiple SABB strains would show synergistic or additive effects on the fitness of soybean plants collectively. SABB mixtures were formulated with SABB strains that are compatible with each other based on the co-culture compatibility assay (**Supplementary Table S2**). We used *Bacillus*, *Pseudomonas*, and *Enterobacter* strains as base strains because of their strong antifungal and other beneficial activities. The SABB mixtures tested were composed of 5-23 selected SABB strains (**Supplementary Table S3**; **Supplementary Figure S3**), and from this point we use the term ‘synthetic bacterial community (SBC)’ for the SABB mixtures used in this study.

### 3.2 Effect of SBCs on soybean growth and yield

**Figure 2:**
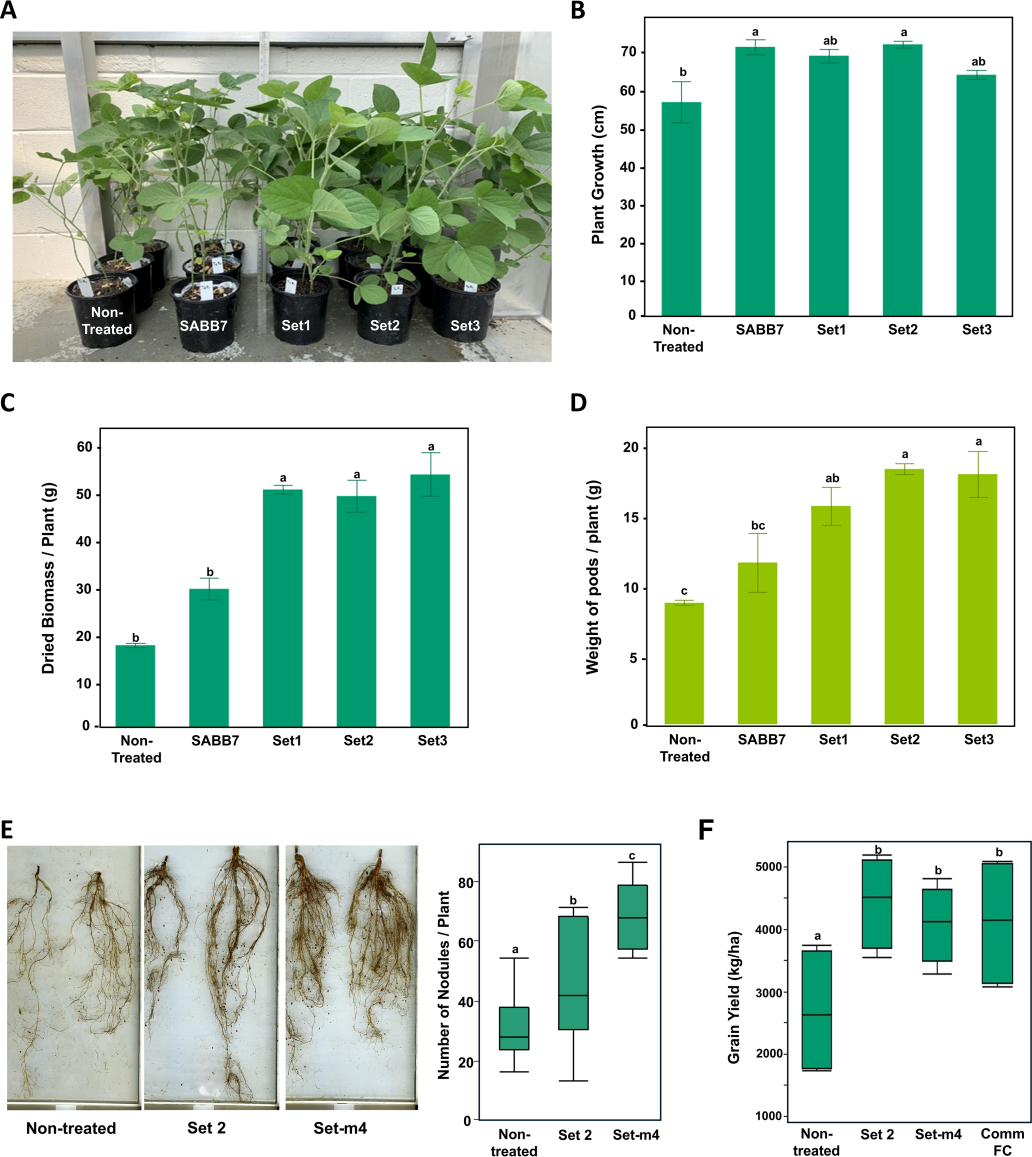
Effects of SABB consortia on the growth and yield of soybean plants in greenhouse **(A–E)** and field **(F)** conditions. **(A)** Representative soybean plants of the different treatments at 28 days after planting. **(B)** Plant growth at R1 Stage. **(C)** Biomass in dry weight per plant at harvest. **(D)** Biomass of pods per plant in dry weight at harvest. **(E)** Images of representative roots with nodule formation and the number of root nodules per plant. **(F)** Grain yield in the soybean field. Letters on the bar and box plots represent significant differences based on Tukey’s HSD tests at 5% probability level. Error bars indicate standard errors of the means from four and five replications in the greenhouse and field experiments, respectively. Comm FC, Commercial fungicide (Apron Maxx RTA, Syngenta).

In greenhouse trials using the natural field soil from the field at the Doyle Chambers Central Research Station (Baton Rouge, Louisiana, USA), all three initial synthetic bacterial communities (SBCs), Set-1, Set-2, and Set-3, demonstrated significant growth promotion in biomass and yield (**Supplementary Table S3**; **Supplementary Figure S4**; **Figures 2A–D**). Subsets of Set1, Set2, and Set3: Setm1 through Setm4, were also evaluated to get minimal sets having maximum efficacy, and Setm4 was chosen along with Set2 for further analyses due to its highest growth-promoting efficacy among the four subsets tested (**Supplementary Figure S4**).

Nodulation assessments using a WinRHIZO root scanner (Regent Instruments Inc., Canada) revealed that Set2- or Setm4-treated plants showed a significantly greater number of nodules compared to the non-treated group, with Setm4 being more effective than Set2 in promoting nodule formation (**Figure 2E**). The number of nodules per plant showed a significant correlation with overall plant growth (Spearman correlation rs= 59%, *p* = 0.0103). In the field condition, plants grown from seeds treated with Set2, Setm4, or a commercial seed-treating product (Comm FC) exhibited significantly higher yields (*p* < 0.05) than those from untreated seeds (**Figure 2F**).

### 3.3 Effect of SBCs on disease resistance to *R. solani*

**Figure 3:**
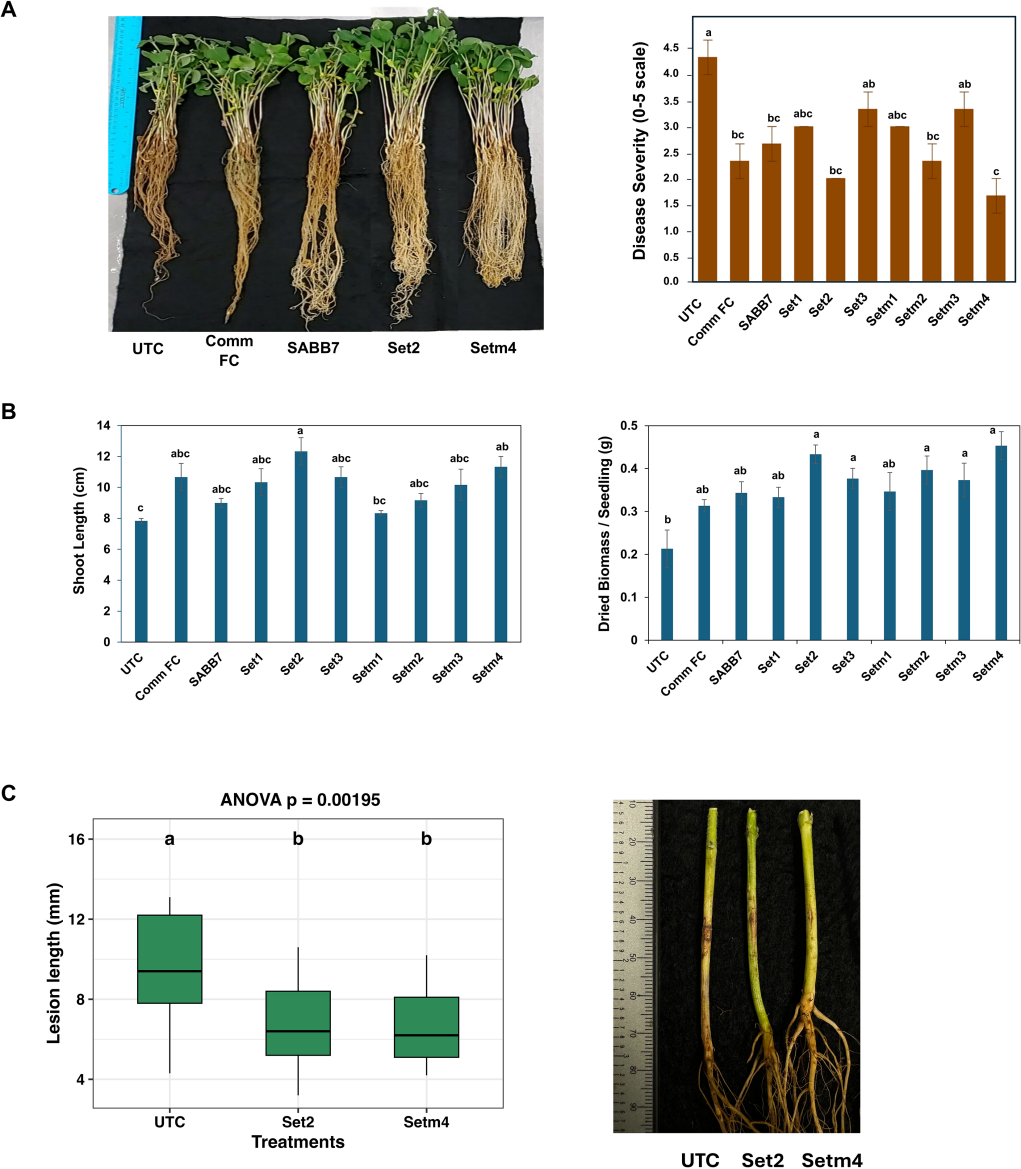
Effects of the seed treatments with soybean-associated beneficial bacteria on soybean plants inoculated with the fungal pathogen *Rhizoctonia solani*. **(A)** Effects of SBCs on soybean seedlings inoculated with *Rhizoctonia solani*. ‘SABB7’ indicates the seed treatment with the single SABB strain (*Bacillus* sp.) only, and disease severity was assessed at 7 days post-inoculation (dpi). The bar graph shows the severity of crown and root rot lesions caused by the pathogen 7 dpi in growth chamber conditions (n = 3 replications). **(B)** Effect on the seedling growth in an infected condition in the growth chamber, assessed 14 days after planting (dap). Seedling growth parameters (shoot length and seedling dry weight) were measured 14 dap (n = 3 replications). **(C)** Disease severity of soybean plants inoculated with *R. solani* in greenhouse conditions with representative infected seedlings photographed after 7 dpi (n = 15 replications). ANOVA followed by Tukey’s HSD test (*p* < 0.05) was used to determine significant differences among treatments. UTC, untreated control; Comm FC, commercial fungicide (Apron Maxx RTA, Syngenta).

The SBCs tested for growth-promoting activities, Set1 to Set3 and Setm1 to Setm4, were also further evaluated under controlled growth chamber conditions for their efficacy in defense enhancement through inoculation with the fungal pathogen *Rhizoctonia solani* (**Figures 3A, B**). Soybean seedlings were inoculated at the cotyledon or VC stage (4 days after emergence). Among all treatments, Set2 and Setm4 significantly enhanced seedling growth by 45 – 57% with two-fold increase in biomass (*p* < 0.05) and improved root development with reduced root rot lesions by 53 – 61% compared to the untreated control (*p* < 0.0001) (**Figures 3A, B**). Suppression of lesion development by Set2 and Setm4 treatments was also observed in additional greenhouse experiments (**Figure 3C**). No significant difference was observed between Set2 and Setm4, indicating that both seed treatments were equally effective in suppressing lesion development caused by *R. solani* (**Figure 3C**).

### 3.4 Effects of bacterial seed treatments on soybean development under abiotic stresses

**Figure 4:**
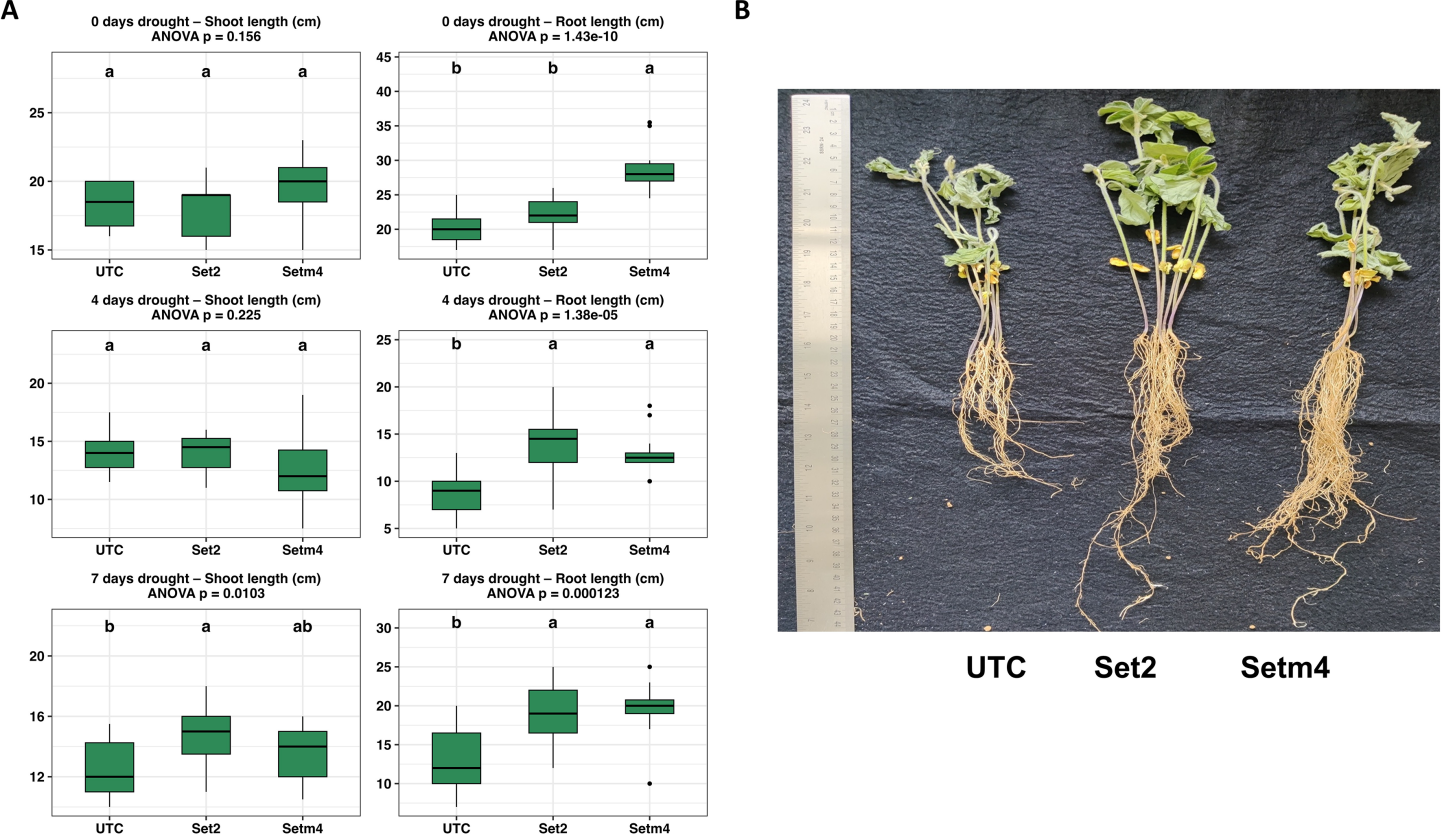
Effects of synthetic bacterial community (SBC) treatments on soybean seedling growth under drought stress. **(A)** Shoot and root lengths of soybean plants in different drought conditions (n = 15 replications). **(B)** Representative seedlings photographed after 7 days of no-watering (drought) period. No-watering conditions for drought stress were imposed on soybean plants 3–4 days after emergence. ANOVA followed by Tukey’s HSD test (*p* < 0.05) was used to determine significant differences among treatments. UTC, untreated control.

Growth responses of soybean plants to drought stress were evaluated in terms of shoot and root development across Set2, Setm4, and untreated controls as shown in **Figure 4**. Before imposing drought condition (0 days of drought), SBC treatments resulted in no significant differences in shoot length (p = 0.156), while Setm4-treated seedlings exhibited a significantly longer root system than controls (p < 0.001) (**Figure 4A**). Under moderate drought stress (4 days), shoot lengths remained similar across treatments (p = 0.225). In contrast, root development was enhanced significantly in seedlings treated with Set2 and Setm4 compared to untreated controls (p < 0.001) (**Figure 4A**). Under a prolonged drought condition (7 days), effects of SBC treatments became more pronounced. Set2- and Setm4-treated seedlings showed significantly longer shoot length (p = 0.010) and root length (p < 0.001) compared to untreated controls, which were nearly collapsed by dryness (**Figures 4A, B**). Effects of SBC treatments on drought tolerance were also supported by the biomass data of individual plants in fresh and dry weights (**Supplementary Figure S5**).

**Figure 5:**
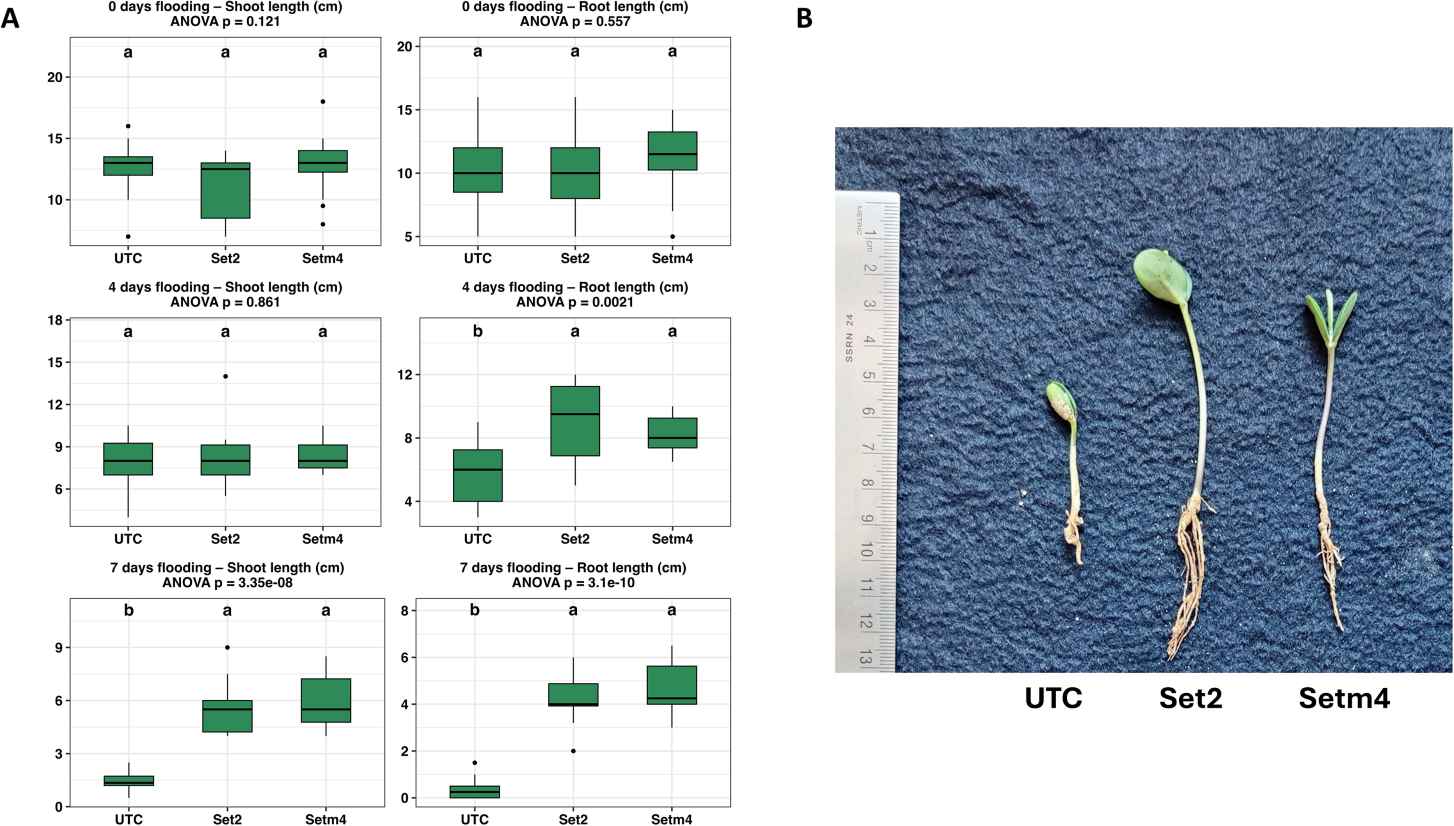
Effects of bacterial seed treatments on soybean seedling growth under flooding stress imposed immediately after emergence. **(A)** Shoot and root lengths of soybean plants in different water-logging conditions (n = 12 replications). **(B)** Representative seedlings photographed after 7 days of waterlogging. Water-logging conditions for flooding stress were imposed on soybean plants 3–4 days after emergence. ANOVA followed by Tukey’s HSD test (*p* < 0.05) was used to determine significant differences among treatments.

Growth responses of soybean plants to flooding stress were also evaluated in terms of shoot and root development across Set2, Setm4, and untreated controls as shown in **Figure 5**. Before imposing a flooding condition (0 day), bacterial treatments showed no significant differences in shoot length (p = 0.121), and root length differences were also not significant (p = 0.557) (**Figure 5A**). Under moderate flooding stress (4 days), shoot lengths remained similar across treatments (p = 0.861) (**Figure 5A**). However, root length was significantly enhanced in both Set2- and Setm4-treated seedlings (p = 0.002), with bacterial treatments supporting nearly 40-50% greater root elongation than controls (**Figure 5A**). Under prolonged flooding stress (7 days), both shoot and root lengths were significantly improved in Set2 and Setm4-treated plants (p < 0.001 for both), with roots nearly 10 to 11 times longer than controls (**Figures 5A, B**). Furthermore, dry weight data of the plants under the 5-day-water-logging condition also revealed positive effects of SBC treatments on waterlogging tolerance (**Supplementary Figure S6**).

### 3.5 Characterization of the bacterial community structure and core microbiota associated with SBC-treated plants

**Figure 6:**
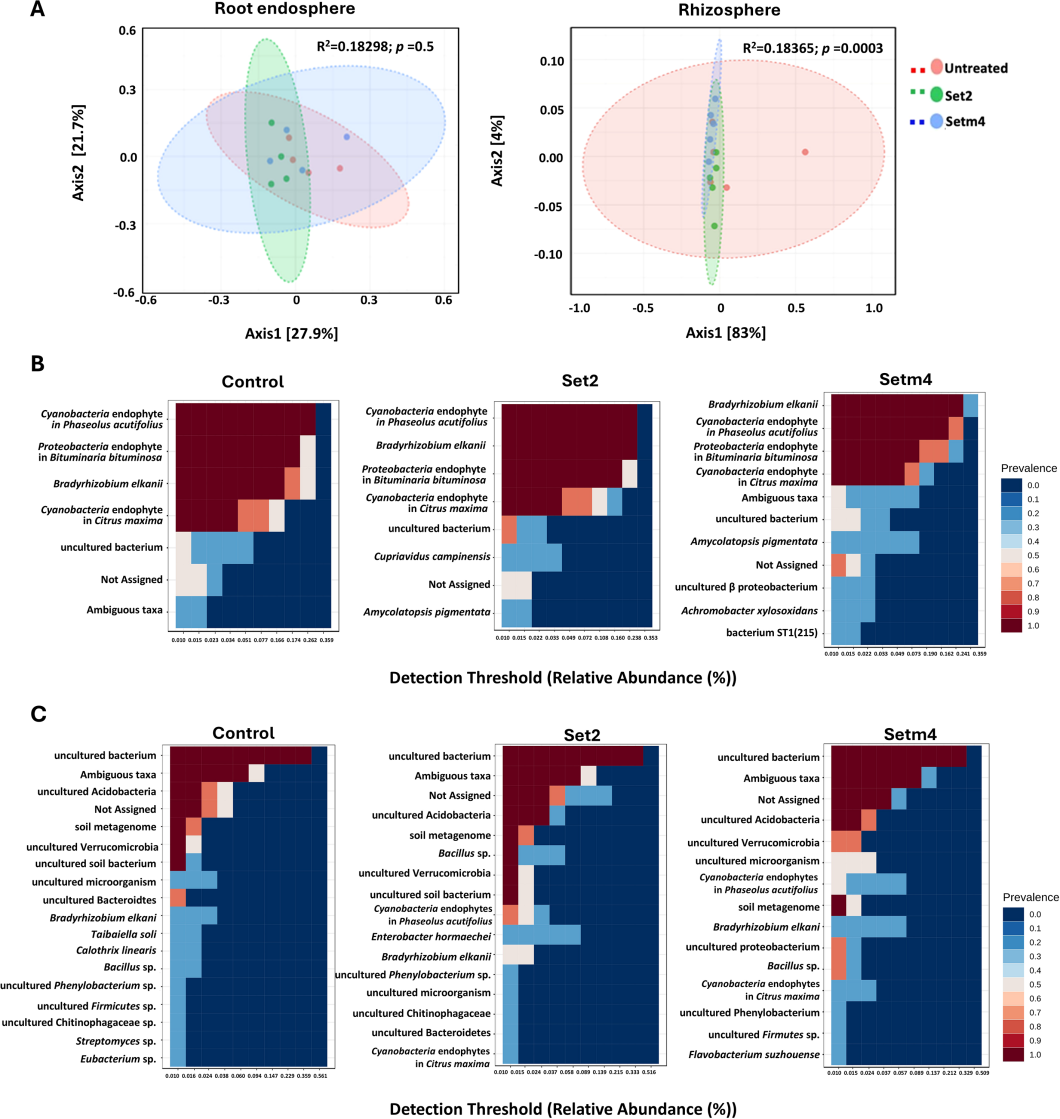
The microbiota structures of the root endosphere and rhizosphere compartments from SBC-treated soybean plants. **(A)** PCoA plots representing the beta diversity among in the endosphere and rhizosphere compartments. **(B)** Heatmaps of taxa representing the core microbiota in the root endosphere. **(C)** Heatmaps of taxa representing the core microbiota in the rhizosphere. Y-axis represents the prevalence level of taxa (core features) across the detection threshold (relative abundance) range on X-axis. The species names shown on the Y-axes in panels **(B, C)** represent the closest matches in the reference database based on 16S rRNA gene sequence similarity; they do not necessarily reflect the actual taxonomic identity at the species level.

The Illumina MiSeq sequencing yielded a total of 3,641,336 reads for both the root endosphere and rhizosphere compartments (**Supplementary Table S4**). The microbial community of soybean plants grown in a Cancienne silt loam field soil, under the greenhouse condition, exhibited no significant variations in alpha diversity metrics among treatments. Both species richness based on observed features and Shannon diversity reflecting richness and evenness did not vary significantly, as indicated by Kruskal-Wallis non-parametric tests (*p* > 0.05; **Supplementary Table S5**). Although there was no noticeable change in the beta diversity of the root endosphere, the rhizosphere microbial community structure showed distinct patterns in seed treatments compared to the non-treated control (**Supplementary Table S5**). The principal coordinate analysis (PCoA) revealed that seed treatments had a marginally significant effect on the rhizosphere microbial community structure and composition (**Figure 6A**). Permutational ANOVA (PERMANOVA) based on Bray Curtis dissimilarity showed a significant difference among treatment groups (**Supplementary Table S5**, **Figure 3A**, p = 0.0003, R^2^ = 0.18365). However, permutational analysis of multivariate dispersions (PERMDISP) analysis revealed a significant difference in dispersion, indicating heterogenous variance within treatments (*p* < 0.05). This indicates that the significant PERMANOVA is not attributed to a significant shift in the mean community structure. Both Set2 and Setm4-treated samples converged toward a more uniform and less variable community, indicating that it streamlined the rhizosphere microbiota e into a more predictable state without fundamentally altering the microbial community structure (**Figure 6A**).

The core root endosphere microbiota was primarily composed of *Bradyrhizobium* sp., along with three endophyte taxa commonly associated with *Phaseolus acutifulus*, *Citrus maxima*, and *Bituminaria bituminosa*, which belong to the phyla Cyanobacteria or Proteobacteria, as well as an uncultured bacterium. The core microbiota maintained a consistent composition within the endosphere compartment; however, seed-treated plants exhibited a higher number of core taxa compared to untreated plants (**Figure 6B**). *Bradyrhizobium* sp., a nitrogen-fixing symbiont of soybeans, showed a significantly higher abundance in seed-treated plants, especially in Setm4 treatment. In this treatment, the prevalence of the bacterium reached 30% at a detection threshold of 0.359, which increased to 100% at a lower threshold of 0.241. Similarly, Set2 treatment also displayed 100% prevalence at a detection threshold of 0.238, while the untreated control had the same prevalence at a lower threshold of 0.166. The high prevalence values at higher detection threshold levels indicate that *Bradyrhizobium* sp. was promoted in the root endosphere of seed-treated plants (**Figure 6B**). *Achromobacter* sp., identified as a distinct core taxon in SBC-treated plants (Setm4), demonstrated a prevalence of 30% at a detection threshold of 0.022 (**Figure 6B**). Other notable taxa included *Cupriavidus* sp. and *Amicolatopsis* sp., which were detected only in SBC-treated plants although they were not components of SBCs. *Cupriavidus* sp. emerged as a distinct core taxon in Set2, with a prevalence of 30% at a detection threshold of 0.033. A*. pigmentata* was present in SBC-treated plants, showing 30% prevalence in both Set2 (detection threshold: 0.015) and Setm4 (detection threshold: 0.073), but was absent in the non-treated control (**Figure 6B**).

In the rhizosphere microbiota, fourteen core taxa were identified across the treatments. These primarily belonged to the phyla Acidobacteria, Verrucomicrobia, Bacteroidetes, and Firmicutes (**Figure 6C**). Notably, *Bradyrhizobium* sp. was consistently found in a significant proportion of rhizosphere samples, with prevalence rates ranging from 30 to 50%. In Set2 treatment, *Bradyrhizobium* sp. exhibited a prevalence of 50% at a detection threshold of 0.015. Both Setm4 and the untreated control showed a prevalence of 30%, although the detection threshold was higher in Setm4 (0.057) compared to the untreated control (0.024). Additionally, *Bacillus* sp. showed a prevalence of 100% and 70% at a detection of 0.01 in Set2 and Setm4 treatments, respectively, while *Enterobacter* sp. did a prevalence of 30% at a detection threshold of 0.058 (**Figure 6C**). In contrast to the core microbiota of the root endosphere, the untreated group’s rhizosphere contained four distinct core taxa that were not detected in the two SBC-treated groups. These unique core taxa included *Eubacterium* sp.*, Calothrix linearis* sp., *Streptomyces* sp., and *Taibaiella* sp. (**Figure 6C**).

### 3.6 Persistence of SABB seed treatment strains, differential abundance, and prediction of significant species in the microbial communities through co-occurrence network analysis

**Figure 7:**
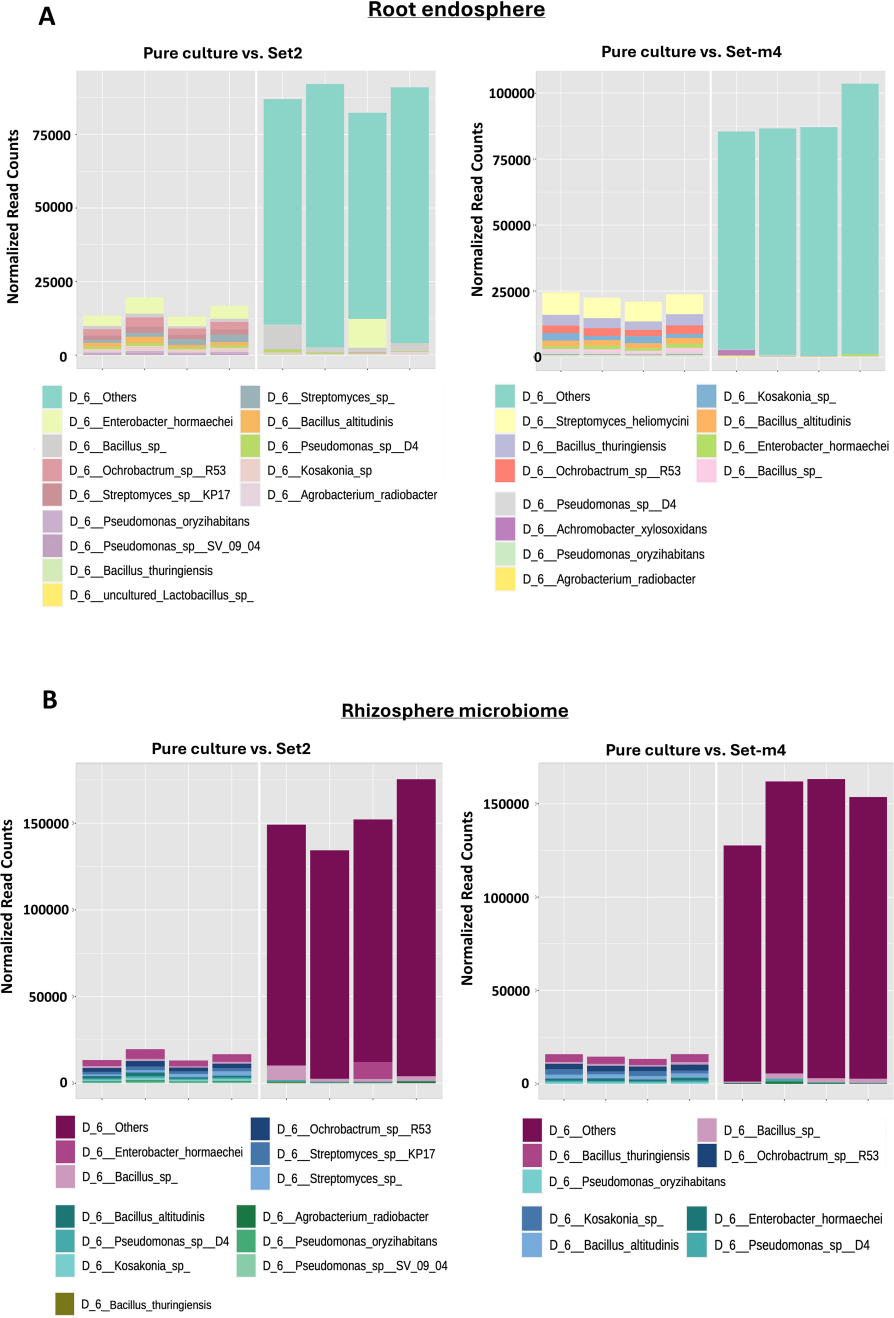
Sequence read comparisons of SBC components for Set2 and Setm4 between their pure culture mixtures and the associated root endosphere **(A)** and rhizosphere **(B)** microbial communities at the species level. Microbiota profiles in the root endosphere and rhizosphere were determined 60 days after planting and sequence reads that do not match any of the SBC components were classified as ‘Others’. The species names in panels A and B represent the closest matches in the reference database based on 16S rRNA gene sequence similarity; they do not necessarily reflect the actual taxonomic identity at the species level. Scientific names embedded within the figure are not italicized because their font style is fixed in the output images.

Within the SABB strains encompassing Set2 and Setm4, a total of five SABB strains were detected in the root endosphere based on the sequence reads at 60 days after planting in the mesocosm study. *Achromobacter* sp., exclusive to Setm4, was detected solely in the plants treated with Setm4, while *Agrobacterium* sp., specific to Set2, was exclusively found in Set2-treated plants. The other SABB strains detected in the root endosphere of the SBC-treated plants were *Enterobacter* sp.*, Pseudomonas* sp. D4, and *Bacillus* sp. (**Figure 7A**). In the rhizosphere microbiota, nine strains of the seed-treated SBCs were detected. Both Setm4-treated and Set2-treated plants contained *Bacillus* sp., *Pseudomonas* sp., and *Enterobacter* sp. *Achromobacter* sp. was exclusively found in Setm4-treated plants, while *Agrobacter* sp.*, Streptomyces* sp.*, Pseudomonas* sp., and *Orchobactrum* sp. were present in Set2-treated plants (**Figure 7B**). Seed treatments with Setm4 and Set2 also resulted in enriched (3 to 9 log_2_ fold changes) and depleted ASVs (-3 to -10 log_2_ fold changes), indicating significant alterations in the root-associated microbiota (**Supplementary Table S6**).

**Figure 8:**
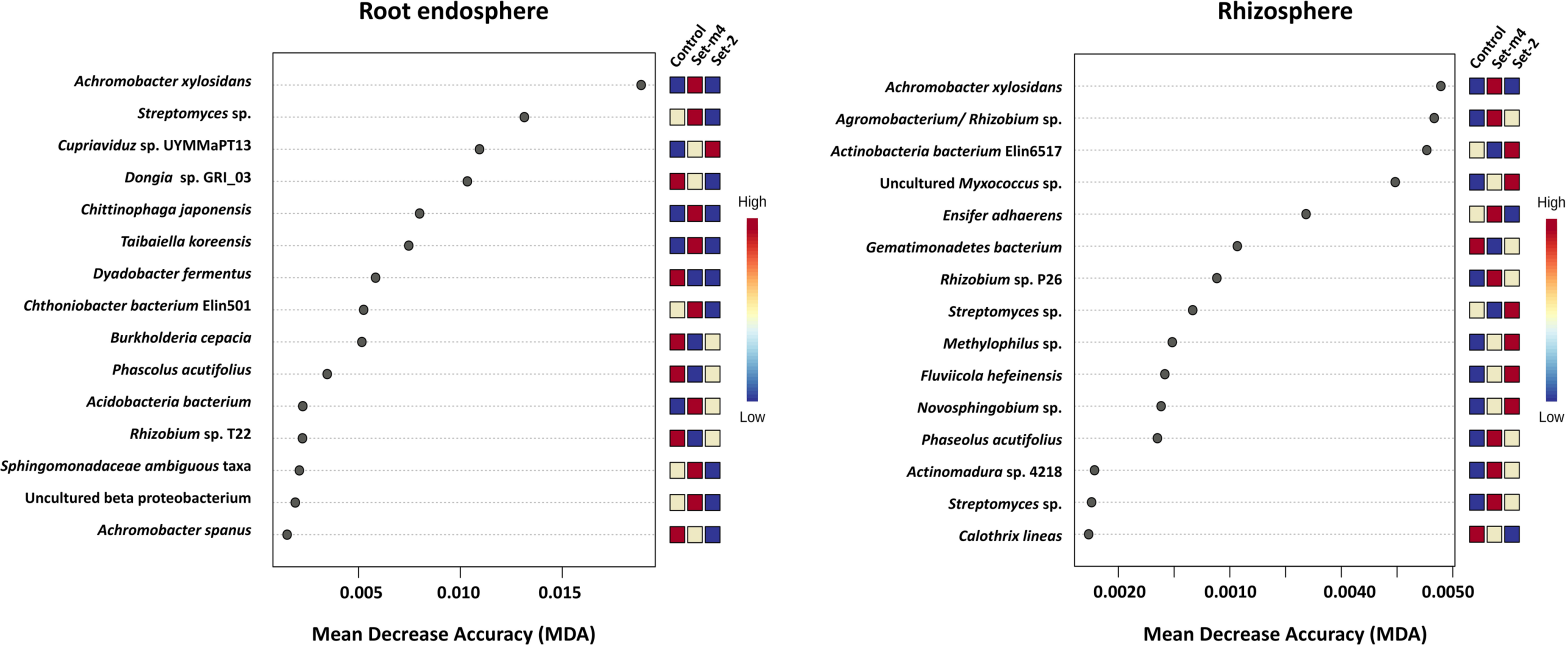
Significant species identified by Random Forest in the root endosphere and rhizosphere microbiomes. The features are ranked by the mean decrease accuracy based on permutation, and mini heatmaps visualize the patterns of change across the untreated control and Setm4 and Set2 treatments. The species names shown on the Y-axes in panels represent the closest matches in the reference database based on 16S rRNA gene sequence similarity; they do not necessarily reflect the actual taxonomic identity at the species level.

The strains of the seed-treated SBCs identified as significant taxa using Random Forest (RF) algorithm were *Achromobacter* sp. and *Streptomyces* sp. in the root endosphere microbiome, while *Achromobacter* sp.*, Agrobacterium/Rhizobium* sp.*, Ensifer* sp., and *Streptomyces* sp. in the rhizosphere (**Figure 8**).

**Figure 9:**
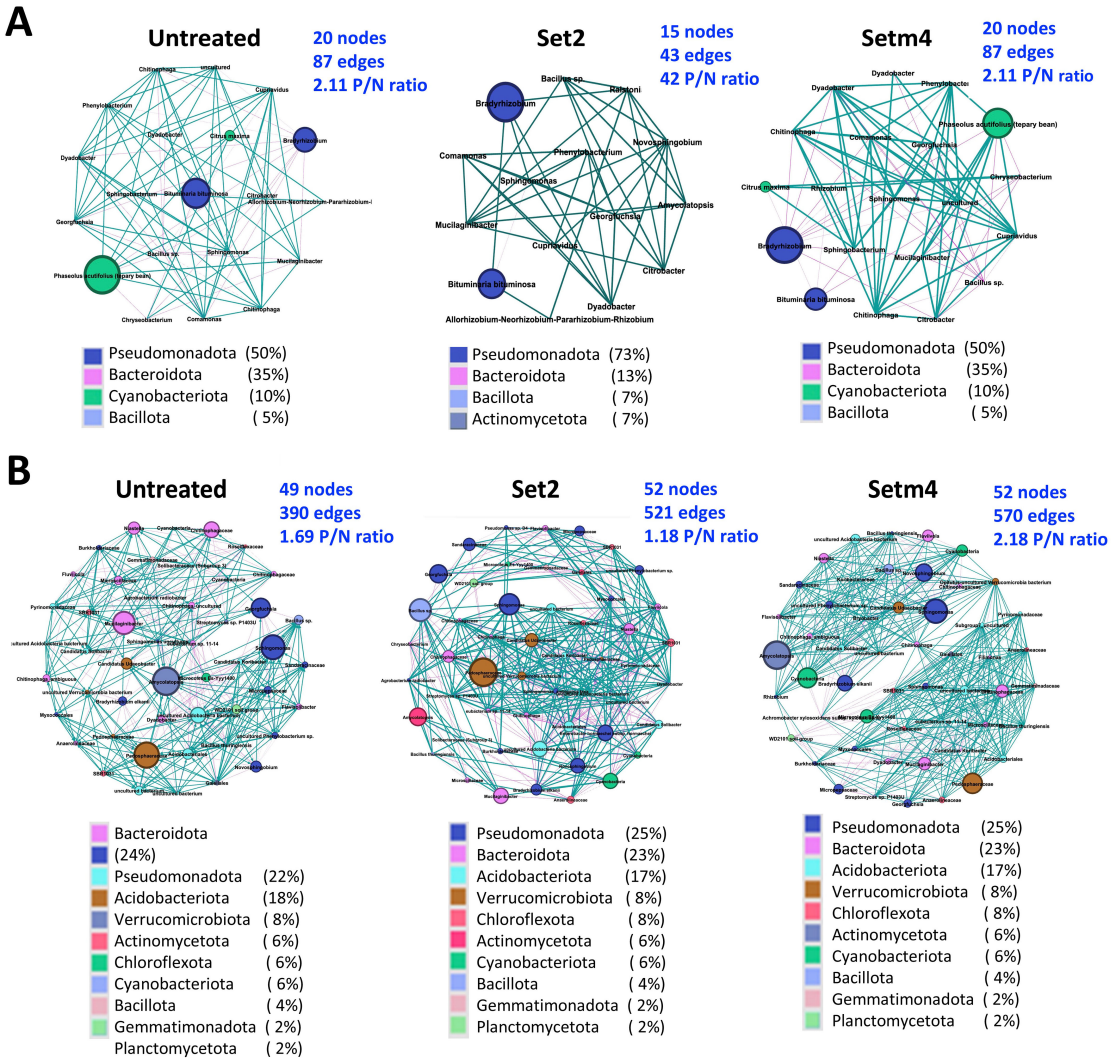
Co-occurring taxa revealed in microbial interaction networks of dominant taxa in microbiota of the endosphere (top 35 taxa) **(A)** and the rhizosphere (top 55 taxa) **(B)**. Only taxa with significant correlation become nodes and the size shows the abundance, and the different colors indicate the corresponding taxonomic assignment at the phylum level. The edge color represents positive (green) and negative (pink) correlations. The edge thickness indicates the correlation values; only significant interactions are shown (r > 0.6; *p* < 0.01). The percentages shown in parentheses represent the proportion of taxa belonging to each phylum. Key network topology metrics (number of nodes, number of edges, ratio of positive to negative correlations (P/N ratio) are indicated next to each network. Scientific names embedded within the figure are not italicized because their font style is fixed in the output images.

Additionally, we identified key taxa associated with seed treatments based on the microbial co-occurrence network using the PageRank algorithm. The key taxa associated with soybean endophytic roots based on relative abundance were *Bradyrhizobium* sp. and endophytic bacteria of *Phaseolus acutifolius* (*Cyanobacteriota*) and *Bituminaria* sp. (*Alphaproteobacteria*) across all the treatments (**Figure 9A**). *Bradyrhizobium* sp. was more abundant in the SBC-treated endospheres (**Figure 9A**).

In the rhizosphere, the seed treatment strains were co-present with known beneficial bacteria belonging to *Rhizobiales*, such as *Bradyrhizobium* sp*, Mesorhizobium* sp., and *Rhizobium* sp. (**Figure 9B**). The characterized bacterial community structure using a co-occurrence network analysis indicated a nonrandom co-occurrence pattern in both compartments (**Supplementary Table S7**). The microbial community showed no significant differences in network complexity between the SBC-treated and the untreated plants in the root endosphere. However, the network in the rhizosphere was denser and highly connected in the Set2- or Setm4-treated plants than in the untreated plants (**Figure 9B**). Set2 had higher connectivity, but lower P/N ratio than the untreated (PN ratio: 1.18 Set2 < 1.69 UTR), while Setm4 exhibited highly connected networks with P/N ratio of 2.18, which indicates a community dominated by positive co-occurrence patterns. These shifts in key network topologies point to a treatment-specific restructuring of microbial interaction patterns, as reflected by the higher number of interacting nodes, edges, average clustering coefficient, average path length, and the greater number of modules or bacterial groups (**Supplementary Table S8**).

## 4 Discussion

In this study, we identified more than 300 SABBs based on diverse microbial traits related to soybean growth and health, including solubilization of plant nutrients, production of plant growth hormones, and suppression of plant pathogens (Lugtenberg and Kamilova, 2009; Glick, 2012; Glick and Nascimento, 2021; Bloemberg and Lugtenberg, 2001; Santoyo et al., 2016; Rashid et al., 2012). While each single SABB showed a marginal effect, synthetic bacterial communities (SBCs) composed of multiple SABBs were significantly effective in suppressing seedling root rot in the lab assay and higher yield in the field conditions. This points to a collective functionality of the SBCs resulting from additive or synergistic effects of their components.

The biocontrol potential of the SBCs could be attributed to the SABB strains belonging to *Bacillus* and *Pseudomonas*, which are known for producing antibiotics, secondary metabolites, and lytic enzymes reported to act against *R. solani* (Cawoy et al., 2015). Other SABB strains of the SBCs belong to the genus *Enterobacter*, *Achromobacter, Rhizobium, Ensifer, Streptomyces, Leclercia*, and *Kosakonia*, which are known as plant growth-promoting rhizobacteria capable of N-fixation, phosphate solubilization, IAA and siderophore production, and/or starch hydrolysis (Ciampitti and Salvagiotti, 2018; Kim et al., 2014; Graham et al., 2016; Palaniyandi et al., 2014). *Bacillus*, *Pseudomonas, Enterobacter* spp.*, Leclercia*, and *Streptomyces* are capable of producing auxin (Shafi et al., 2017; Rolli et al., 2015; Kim et al., 2014; Graham et al., 2016; Palaniyandi et al., 2014), and most soil bacteria can convert insoluble soil P into plant available forms (Alori et al., 2017; Anand et al., 2016). The SABB strains *Bacillus* sp., *Enterobacter* sp., *Pseudomonas* sp., *Rhizobium* sp., and *Ensifer* sp. produced siderophores, which play a significant role in plant iron nutrition and biocontrol of soil-borne plant diseases (Lugtenberg and Kamilova, 2009). *Enterobacter* sp., *Pseudomonas* sp., *Bacillus* sp., and *Leclercia adecarboxylata* can also hydrolyze starch, which indicates their ability to produce amylase and oligo-1,6-glucosidase for nutrient mineralization in the soil (Pascon et al., 2011).

The overlapping beneficial traits of the SABBs comprising the SBCs in this study may confer broader and stronger impacts on plant growth and protection compared to those of a single SABB. Meta-analysis of microbial biodiversity-ecosystem functioning conducted by Saleem et al. revealed that higher proportion of underlying mechanisms of microbiome functions were complementarity/synergy and functional redundancy (Saleem et al., 2019). Recent work by Wicaksono et al. (2024) supported the strong functional redundancy in plant rhizosphere microbiome, while other studies demonstrated that consortia of multiple beneficial strains outperform single strains by combining distinct functions that stabilize plant fitness under field conditions (Moretti et al., 2024; Wang et al., 2024).

The enhanced soybean resistance to the fungal pathogen *R. solani* by the seed treatment of SBCs strongly suggests its ‘defense-priming’ effect on soybean plants, which makes them induce more robust and rapid defense responses upon pathogen infection compared to untreated plants. We are conducting RNA sequencing analyses to test this hypothesis and to identify defense pathways involved in the SBC-induced disease resistance.

In this study, the SBCs (Set2 and Setm4) also improved soybean tolerance to drought and waterlogging conditions. The most consistent response across stresses was root system enhancement, which plays a central role in stress adaptation. Longer and healthier roots allow greater access to oxygen in waterlogged or flooded soils and deeper water absorption under drought, giving treated plants a physiological advantage over untreated controls. These positive impacts of SBCs on the tolerance to the abiotic stresses can be explained by well-documented functions of plant growth-promoting rhizobacteria (PGPR) because both SBCs contain bacterial species known to be PGPR.

PGPR are known to modulate hormone balance through the production of auxins, gibberellins, and abscisic acid, which collectively regulate root and shoot architecture, stomatal function, and stress signaling under abiotic stresses (Kumar et al., 2016; Zhang et al., 2022). Particularly, some PGPR are capable of producing 1-aminocyclopropane-1-carboxylate (ACC) deaminase, which could lower stress-induced ethylene levels and prevents premature root senescence under both flooding and drought as previously reported (Maxton et al., 2017; Vandana et al., 2024). Antioxidant enzymes stimulated by PGPR, including catalase, peroxidase, and superoxide dismutase, are also known to help alleviate oxidative damage associated with hypoxia during flooding and dehydration during drought (Arya et al., 2021; Gowtham et al., 2022; Moretti et al., 2020; Nivetha et al., 2021). In addition, PGPR may induce the expression of genes linked to anaerobic metabolism, such as alcohol dehydrogenase and pyruvate decarboxylase, which enable plants to generate energy under hypoxic conditions (Komatsu et al., 2021). Proteomic studies further showed that PGPR-treated plants upregulate stress-responsive proteins, such as heat shock proteins and aquaporins, maintaining cellular balance and water status during flooding stress (Guo et al., 2017).

Another important mechanism of enhanced stress tolerance by PGPR is the improvement of soil-plant interactions. Exopolysaccharide production by PGPR enhances soil aggregation and water retention, buffering plants against drought, while also maintaining oxygen diffusion during excess soil moisture conditions (Khan and Bano, 2019). PGPR also promote osmolyte accumulation, such as proline, which stabilizes proteins and membranes and preserves turgor pressure under water deficit conditions (Agami et al., 2016). Enhanced photosynthetic rates and stomatal conductance in PGPR-treated plants have also been reported, supporting their role in maintaining metabolic activity under drought (Chieb and Gachomo, 2023).

Persistence of the seed-treated SABB strains at the vegetative stage was confirmed based on sequence readouts obtained from 16S rRNA gene amplicon sequencing of the pure culture against the microbiome sequence. The observed survival of the seed-treated SABB strains in both endospheric and rhizospheric parts of the soybean rot system implies their positive interactions with the core microbiota and recruitment by the plant as beneficial partners. Nevertheless, the untreated control plants had a higher number of core taxa in the rhizosphere microbiome compared to the SBC-treated plants. This difference was reflected in the beta diversity PCoA plot, showing a significantly narrower cluster for the SBC-treated rhizospheres compared to the untreated rhizosphere. These findings suggest that the SBC seed treatments may modulate the existing community. This may be driven by niche occupancy or competition for nutrients, which leads to a reduced species composition and the restriction of other species in the rhizosphere (Lugtenberg and Kamilova, 2009). This result aligns with an earlier study conducted on founding taxa during the early plant stage of *Arabidopsis thaliana*, which demonstrated their long-lasting impact on microbial community assemblies and their ability to resist invasion by latecomers in the phyllosphere microbial community structure (Carlström et al., 2019). Other studies trying to predict community assembly found that plant-associated microbiota can be affected by the arrival order, also called the ‘priority effect’, in both assembly and function (Fitzpatrick and Schneider, 2020; Wei et al., 2019).

The SABB strains of the SBCs were also identified as significant taxa using the Random Forest model. However, our microbiome sequence data revealed relatively low to slightly moderate accuracy and confidence in predicting species classification between the seed treatments and the untreated samples. While the RF model has demonstrated success in integrating species abundance for phenotypic prediction in the human microbiome, it has downside in identifying consistent patterns between species abundance and phenotypic outcomes in highly diverse soil microbiomes (Blum et al., 2019). These challenges contribute to the observed low and slightly moderate accuracy levels of 40% in the rhizosphere and 60% in the root endosphere. This finding emphasizes the need for further research of more sophisticated modeling approaches that can effectively address the complexities of root-soil microbiomes.

Earlier studies attribute crop productivity as a function of interaction with and among diverse organisms associated with the crop (Poudel et al., 2016; Agler et al., 2016; van der Heijden and Hartmann, 2016). In our study, we found that microbial communities associated with soybean growth exhibited a dense network structure, which indicates a more complex interaction within the rhizosphere. This is consistent with recent findings. Ren et al. (2025) showed that more complex rhizosphere networks correlated with higher soybean yield across diverse agroecosystems. Similarly, Bandara et al. (2021)reported that, between high- vs low-yield fields, the high-yield soybean field sites displayed more complex microbial networks compared to the low-yield sites. Within the SBC-treated microbial network, a non-random co-occurrence pattern was inferred, suggesting cooperative interactions between the seed treatment strains and other key species. Notably, the seed-treated strains showed co-occurrence with *Bradyrhizobium* sp., a nitrogen-fixing bacterium symbiotically associated with soybeans in tropical regions. Furthermore, *Bradyrhizobium* sp. was more abundant in the seed-treated plant compartments. These findings corroborate with an earlier study that linked higher productive soybean field to an increased abundance of *Bradyrhizobium* (Chang et al., 2017).

## 5 Conclusions

Plant-associated beneficial bacteria are known to support plant growth and health, but their simultaneous effects on the associated microbial communities remained poorly explored. With 16S rRNA gene sequence metabarcoding, we showed that seed-treated SABB strains persisted in the microbial communities and co-occurred with symbiotic key taxon, *Bradyrhizobium* sp. These findings provide valuable insight into targeted crop management strategies through the application of bacterial inoculants, although their positive effects on host plants in various natural settings still need to be studied further. Taken together, this study not only suggests the seed treatment of SBC as an ideal direction for the practical use of beneficial bacteria for more sustainable cultivation of soybeans but also the scientific basis underlying the positive effects of this crop management practice.

## Data Availability

The sequence data analyzed in this study were deposited in the NCBI database under the BioProject accession number, PRJNA1181146 (https://www.ncbi.nlm.nih.gov).
