# Supplementary material for: Growth promotion and stress tolerance of soybean plants driven by seed treatment with synthetic bacterial community of soybean-associated beneficial bacteria
Source: Front Plant Sci. 2026 Jan 7;16:1729743. doi: 10.3389/fpls.2025.1729743 (PMC12819778; doi:10.3389/fpls.2025.1729743)
Supplement: Supplementary file 1 [file DataSheet1.pdf]

frontiers

Supplementary Material

1 Methodology

1.1 Screening of antifungal activity against *Rhizoctonia solani*

The antagonistic abilities of the bacterial isolates were determined by dual-plate confrontation assays as previously described (Shrestha et al. 2016). A 5mm diameter mycelial plug from *Rhizoctonia solani* cultured on PDA was placed on the middle of a potato dextrose agar (PDA) plate. A 10 µl suspension of bacterial strain grown in Luria broth (LB) (10 g tryptone, 10 g NaCl and 5 g yeast extract per L) overnight and adjusted to OD_{600} = 0.1 was spot inoculated surrounding the mycelial plug at the center of the PDA plates. The plates were incubated at 30±2 °C and fungal growth was observed after 72 h. Inhibition zones surrounding the colonies were measured and recorded as an indicator of antifungal activity. Then, the bacteria with the most potent inhibitory activity were selected and identified based on their 16S rRNA gene sequences.

1.2 *In vitro* screening of SAB isolates for growth-promoting characteristics

The isolated SABs were tested for their growth-promoting activities including nitrogen fixation, indole-3-acetic acid (IAA) production, phosphate solubilization, siderophore production, and starch hydrolysis.

Nitrogen fixation

Isolates were grown in Jensen's nitrogen-free agar media with bromothymol blue (BTB)(Jensen 1942). The components of Jensen's nitrogen-free agar medium were K_2HPO_4 (1 g/L), $MgSO_4{\cdot}7H_2O$ (1 gL^{-1}), NaCl (1 gL^{-1}), $FeSO_4{\cdot}7H_2O$ (1 gL^{-1}), $Na_2MoO_4{\cdot}2H_2O$ (1 gL^{-1}), $CaCO_3$ (1 gL^{-1}), agar (1 gL^{-1}), and sucrose (1 gL^{-1}) as carbon source(Jensen 1942). The growth of the bacteria in the nitrogen-free medium indicates its ability to utilize atmospheric nitrogen gas for cell protein synthesis.

Indoleacetic acid (IAA) production assay

The isolates were grown in tryptic soy broth with the following formulation (g L^{-1}): $MgSO_4{\cdot}7H_2O$ – 0.15 g, NaCl – 0.15 g, $MnSO_4$ – 0.05 g, $FeSO_4{\cdot}7H_2O$ – 0.05 g, Na_2SiO_3 – 2.0 g, $CaCO_3$ –2.0 g, $Ca_3(PO_4)_2$ – 1.5 g, KNO_3 – 1.0 g, and sucrose – 20 g supplemented with tryptophan (Gordon and Weber 1951). All media used were sterilized for 15 min at 121°C before use. After 7 days of incubation, the cultures were centrifuged at 5000 rpm for 15 min. One milliliter of the supernatant was mixed with 2 ml of Salkowski reagent and the appearance of a pink to red color qualitatively indicates IAA production(Gordon and Weber 1951).

Phosphate Solubilization

Pikovskaya's media with precipitated tricalcium phosphate was used to grow the isolates(Pikovskaya 1948). The bacterial isolates were spot-inoculated using 10 ul of bacterial suspension with OD_{600}=0.1 on the surface of the agar and were incubated for 5 days. Clear or halo zone formation around the bacterial colony indicates phosphate solubilization(Pikovskaya 1948).

Siderophore production

Chrome azurol S (CAS) agar was prepared from 4 solutions, which were sterilized separately before mixing(Alexander and Zuberer 1991). The Fe-CAS indicator solution (solution 1) was prepared by mixing 10 ml of $FeCl_3 \cdot H_2O$ in 10 mM HCl with 50 ml of an aqueous solution of CAS (1.21 mg mL^{-1}). The resulting indicator solution was added slowly with constant stirring, to 40 ml of an aqueous solution of hexadecyltrimethylammonium bromide (HDTMA) (1.82 mg mL^{-1} which was autoclaved and cooled to 50°C. Solution 2 was prepared by dissolving 30.24g of piperazine-N,N'-bis[2-ethanusulfonic acid] (PIPES) in 750 ml of a salt solution containing 0.3g KH_2P0_4, 0.5g NaCl, and 1.0g NH_4Cl. The pH was adjusted to 6.8 with 50% KOH, and water was added to bring the volume to 800 ml,15 g agar was added, and the medium was autoclaved. Solution 3 contained the following: 2 g glucose, 2 g mannitol, 493 mg $MgSO_4 \cdot 7H_2O$, 11 mg $CaCl_2$, 1.17 mg $MnSO_4 \cdot 7H_2O$, 1.4 mg H_3BO_3, 0.04 mg $CuSO_4 \cdot 5H_2O$, 1.2 mg $ZnSO_4 \cdot 7H_2O$ and 1 mg Na_2MoO_4.H2). Solution 3 was autoclaved and cooled to 50°C, then was added to the buffer solution along with 30 ml sterilized 10% (w/v) casamino acids. The indicator solution was added last with sufficient stirring to mix the ingredients. A 10 ul bacterial culture with OD_{600}=0.1 was spot inoculated in the CAS plates. Siderophore-producing bacteria produced an orange halo around the colonies after 24-48 h incubation as positive result.

Starch hydrolysis assay

Bacterial isolates were spot-inoculated on starch agar plates consisting of 3.0 g beef extract, 10.0 g soluble starch, 15.0 g bacteriological agar and 1,000 mL of distilled water(Shruti, Arun, and Yuvnee 2013). After 24 h of incubation, the surface of the plate was submerged with Lugol's iodine solution to determine the formation of starch-iodine complex with the dark brown or black color appearance. Microbial starch hydrolysis was revealed as a clearing zone surrounding bacterial colonies which indicate starch degradation due to production of amylase (Shruti, Arun, and Yuvnee 2013).

2 Supplementary Tables

Table S1. Soil chemical analysis from the mesocosm experiment

Sample	Rep	pH	Total N	Total C	CN ratio	P	K	Ca	Cu	Mg	Na	Su	Zn
Untreated	1	6.27	0.09	0.82	8.79	24.47	80.73	1212.25	1.63	261.79	273.8	8.19	1.16
Untreated	2	6.37	0.08	0.62	7.71	15.19	78.32	1318.86	1.94	283.23	217.4	10.06	0.86
Untreated	3	6.52	0.09	0.55	6.07	23.73	76.6	1196.86	1.55	252.68	278.95	8.54	1.22
Untreated	4	5.95	0.11	0.83	7.75	38.56	74.82	971.83	1.07	208.86	284.48	10.65	1.53
Set2	1	6.07	0.09	0.63	7.43	26.15	80.17	1262.82	1.61	265.19	284.19	9.48	1.34
Set2	2	6.43	0.10	0.79	8.01	33.72	81.97	1211.41	1.56	251.22	276.28	9.45	1.68
Set2	3	6.52	0.09	0.77	8.2	30.07	86.66	1327.85	1.77	283.35	332.03	10.77	1.49
Set2	4	6.46	0.08	0.48	5.82	16.54	71.62	1487.28	2.76	313.56	316.26	12.41	1.15
Setm4	1	6.17	0.09	0.76	8.14	24.39	89.13	1510.76	1.64	279.4	328.43	15.68	1.25
Setm4	2	6.22	0.09	0.61	6.69	29.64	75.34	1225.36	1.71	257.41	235.65	11.36	1.29
Setm4	3	6.00	0.11	0.93	8.76	41.84	75.67	1044.8	1.19	223.33	220.86	9.62	1.63
Setm4	4	6.52	0.09	0.59	6.77	18.96	83.51	1542.17	2.94	328.67	332.65	13.69	1.12

Table S2. Pairwise compatibility among soybean-associated beneficial bacteria (SABB) isolates from the co-culture assay

Isolate	SABB1	SABB2	SABB3	SABB4	SABB5	SABB6	SABB7	SABB8	SABB9	SABB10	SABB11	SAB1-9	SAB2-13	SAB3-21	SAB4-29	SAB5-34	SAB6-47	SAB7-61	SAB8-64	SAB9-81	SAB10-94	SAB11-106	SAB12-108	SAB13-109	SAB14-121	SAB15-171	SAB75	SAB80	SAB214	SAB274	SAB285
SABB1																															
SABB2	+																														
SABB3	+	+																													
SABB4	-	+	n																												
SABB5	+	+	+	+																											
SABB6	+	+	+	n	+																										
SABB7	+	+	+	n	+	+																									
SABB8	-	n	+	n	+	n	+																								
SABB9	+	+	+	n	+	+	+	+																							
SABB10	+	+	+	n	+	+	+	+	+																						
SABB11	-	+	+	+	+	+	+	+	+	n																					
SAB1-9	+	+	+	+	+	+	+	+	+	+	+																				
SAB2-13	+	+	+	+	+	+	+	+	+	n	+	+																			
SAB3-21	-	+	+	+	+	+	+	+	+	+	+	+	+																		
SAB4-29	+	+	+	+	+	+	+	+	+	+	+	+	+	+																	
SAB5-34	+	+	+	+	+	+	+	+	+	+	+	+	+	+	+																
SAB6-47	+	+	+	+	+	+	+	+	+	+	+	n	+	+	+	n															
SAB7-61	+	+	+	+	+	+	+	+	+	+	+	+	+	+	+	+	+														
SAB8-64	+	+	+	+	+	+	+	+	+	+	+	+	+	+	+	+	+	+													
SAB9-81	+	+	+	+	+	+	+	+	+	+	+	+	+	+	+	+	+	+	+												
SAB10-94	+	+	+	+	+	+	+	+	+	+	+	+	+	+	+	+	+	+	+	n											
SAB11-106	-	-	-	+	+	-	-	-	+	-	+	+	+	+	+	+	+	+	+	+	+										
SAB12-108	+	+	+	+	+	+	+	+	+	+	+	+	+	+	+	+	+	+	+	+	+	+									
SAB13-109	+	+	+	+	+	+	+	+	+	+	+	+	+	+	+	+	+	+	+	+	+	+	+								
SAB14-121	+	+	+	+	+	+	+	+	+	+	+	+	+	+	+	+	+	+	+	+	+	+	+	+							
SAB15-171	+	+	+	+	+	+	+	+	+	+	+	+	+	+	+	+	+	+	+	+	+	+	+	+	+						
SAB75	+	+	+	+	+	+	+	+	+	+	+	+	+	+	+	+	+	+	+	+	+	+	+	+	+	+					
SAB80	+	+	+	+	+	+	+	+	+	+	+	+	+	+	+	+	+	+	+	+	+	+	+	+	+	+	+				
SAB174	+	+	+	+	+	+	+	+	+	+	+	+	+	+	+	+	+	+	+	+	+	+	+	+	+	+	+	+			
SAB214	+	+	+	+	+	+	+	+	+	+	+	+	+	+	+	+	+	+	+	+	+	+	+	+	+	+	+	+	+		
SAB285	+	+	+	+	+	+	+	+	+	+	+	+	+	+	+	+	+	+	+	+	+	+	+	+	+	+	+	+	+	+	

* SABB isolate pairwise interaction showing +: compatible, -: incompatible, and n: neutral interactions

Table S3. Composition of the formulated SABB consortia and minimal subsets

SABB Consortia	Number of strains	Isolates	Genera
Set1	9	SABB2, SABB3, SABB4, SABB5, SABB6, SABB7, SABB8, SABB9 & SABB11	*Bacillus & Pseudomonas*
Set2	14	SAB1-9, SAB2-13, SAB3-21, SAB4-29, SAB5-34, SAB6-47, SAB7-61, SAB8-64, SAB9-81, SAB10-94, SAB12-108, SAB13-109, B14-121, B15-171	*Bacillus, Pseudomonas, Enterobacter, Leclercia, Kosakonia, Rhizobium, & Streptomyces*
Set3	23	SABB2, SABB3, SABB4, SABB5, SABB6, SABB7, SABB8, SABB9 & SABB11, SAB1-9, SAB2-13, SAB3-21, SAB4-29, SAB5-34, SAB6-47, SAB7-61, SAB8-64, SAB9-81, SAB10-94, SAB12-108, SAB13-109, B14-121, B15-17	*Bacillus, Pseudomonas, Enterobacter, Leclercia, Kosakonia, Rhizobium (Agrobacterium), & Streptomyces*
Setm1	5	SAB1-9, SAB2-13, SAB4-29, SAB8-64, SAB14-121	*Enterobacter, Pseudomonas & Streptomyces*
Setm2	7	SABB7, SAB1-9, SAB2-13, SAB4-29, SAB8-64, SAB14-121, SAB285	*Bacillus, Enterobacter, Pseudomonas, Streptomyces & Ensifer*
Setm3	9	SABB7, SABB8, SABB9, SABB5, SABB3, SAB2-13, SAB8-64, SAB14-121, SAB285	*Bacillus, Pseudomonas, Streptomyces & Ensifer*
Setm4	10	SABB7, SAB1-9, SAB2-13, SAB4-29, SAB8-64, SAB14-121, SAB75, SAB80, SAB214, SAB285	*Bacillus, Enterobacter, Pseudomonas, Streptomyces, Achromobacter & Ensifer*

[a] Soybean associated beneficial bacteria (SABB) consortia

[b] Number of strains in each consortium

[c] Isolate codes of strains in each consortium

[d] Representative genera in each consortium

Table S4. An overview of the Illumina MiSeq sequencing data for this study

a. Pure culture raw sequence data

File type	Conventional base calls
Encoding	Sanger/Illumina 1.9
Total Sequence	396,246 for 8 samples
GC%	53 -55

- Sequence range: 42,433 – 65,546 sequence reads per sample.

b. Endosphere compartment raw data

File type	Conventional base calls
Encoding	Sanger/Illumina 1.9
Total Sequence	1,222,036 for 12 samples
Sequence length	236-251 (Read 1); 35 -251 (Read 2)
Sequence flag as poor quality	0
GC%	53 -55

- Sequence range: 89,284 – 116,834 sequence reads per sample.

c. Rhizosphere compartment raw data

File type	Conventional base calls
Encoding	Sanger/Illumina 1.9
Total Sequence	2,419,300 for 12 samples
Sequence length	237-251 (Read 1); 35 -251 (Read 2)
Sequence flag as poor quality	0
GC%	52 – 55

- Sequence range: 174,785 – 225,310 sequence reads per sample

Table S5. Alpha and beta diversity metrics of the soybean microbiomes in the root endosphere and rhizosphere compartments

Diversity metrics	Root endosphere microbiome (p-values)	Rhizosphere Microbiome (p-values)
*Alpha diversity		
Observed features	0.442	0.840
Shannon diversity index	0.417	0.309
**Beta diversity		
Bray Curtis	0.130	0.034
Unweighted Unifrac	0.211	0.262
Weighted Unifrac	0.691	0.002

Table S6. The ASVs at the species level enriched/depleted in different seed treated compartments

Root endosphere microbiome				
	Enriched ASVs at the species level	Phylum	Log2 FC	P-adjusted value
Set2 vs UT	ASV23_Cupriavidus sp. UYMMaPT13	Proteobacteria	6.733650671	0.01236993
	ASV80_uncultured Novosphingobium sp.	Proteobacteria	3.901187456	0.05800899
	ASV89_Variovorax sp. VA23	Proteobacteria	3.662923331	0.08031122
	ASV14_Chitinophaga	Bacteroidetes	3.640713308	0.08031122
	ASV131_Terrimonas_uncultured bacterium	Bacteroidetes	3.519053716	0.0789495
	ASV106_Burkholderiaceae uncultured bacterium	Proteobacteria	3.507418987	0.08031122
	ASV64_Fluviicola uncultured bacterium	Bacteroidetes	3.434734643	0.06377703
	ASV165_Streptomyces sp	Actinobacteria	3.372661278	0.08931069
	ASV102_Sediminibacterium sp. XWS-42	Bacteroidetes	3.313531002	0.08031122
	ASV73_uncultured Firmicutes bacterium	Firmicutes	3.30469213	0.08031122
Setm4 vs UT	ASV15_Achromobacter xylosoxidans	Proteobacteria	9.13756285	5.49E-07
	ASV43_Chitinophaga japonensis	Bacteroidetes	6.67126127	0.00036421
	ASV55_Taibaiella koreensis	Bacteroidetes	5.93617604	0.00185099
	ASV51_Lysobacter	Proteobacteria	6.08580608	0.00210857
	ASV23_Cupriavidus sp. UYMMaPT13	Proteobacteria	6.73365067	0.01236993
	ASV57_Terrimonas	Bacteroidetes	5.65211388	0.02708014
	ASV91_Sphingomonadaceae_ambinuous taxa	Proteobacteria	4.53406675	0.02708014
	ASV99_Brivibacillus_uncultured bacterium	Firmicutes	4.34808231	0.05800899
	ASV108_Stenotrophobacter uncultured	Acidobacteria	4.06130142	0.05800899
	ASV80_uncultured Novosphingobium sp.	Proteobacteria	3.90118746	0.05800899
	Depleted ASVs at the species level	Phylum	Log2 FC	P-adjusted value
	ASV27_Sphingobacterium sp.	Bacteroidetes	-7.438833029	0.00675736
	ASV35_Streptococcus sp.	Firmicutes	-7.063404218	0.00921599

Set2 vs UT	ASV48_Burkholderia cepacia	Proteobacteria	-6.114833664	0.00036421
	ASV59_uncultured bacterium	Firmicutes	-5.62607783	0.02708014
	ASV63_Rhizobium sp. T22	Proteobacteria	-5.204206218	0.00342131
	ASV96_Dyadobacter fermentans	Bacteroidetes	-4.60399668	0.01236993
	ASV98_Variovorax	Proteobacteria	-4.416056027	0.02708014
	ASV100_Methylophilus sp. La2-4 HL-2016	Proteobacteria	-4.259466091	0.03363351
	ASV111_Taibaiella soli	Bacteroidetes	-4.23123596	0.02455045
	ASV97_uncultured bacterium	Proteobacteria	-3.801380267	0.06377703
Setm4 vs UT	ASV27_Sphingobacterium sp.	Bacteroidetes	-7.438833029	0.006757358
	ASV35_Streptococcus sp.	Firmicutes	-7.063404218	0.009215987
	ASV48_Burkholderia cepacia	Proteobacteria	-6.114833664	0.000364205
	ASV59_uncultured bacterium	Firmicutes	-5.62607783	0.027080145
	ASV63_Rhizobium sp. T22	Proteobacteria	-5.204206218	0.003421311
	ASV96_Dyadobacter fermentans	Bacteroidetes	-4.60399668	0.012369927
	ASV98_Variovorax	Proteobacteria	-4.416056027	0.027080145
	ASV100_Methylophilus sp. La2-4 HL-2016	Proteobacteria	-4.259466091	0.033633508
	ASV111_Taibaiella soli	Bacteroidetes	-4.23123596	0.024550454
	ASV49_Paenibacillus	Firmicutes	-4.149103019	0.061111101

**Rhizosphere microbiome**				
	Enriched ASVs at the species level	Phylum	Log2 FC	P-adjusted value
Set2 vs UT	ASV509_Methylobacillus_uncultured	Proteobacteria	6.6463821	1.02E-07
	ASV440_Novosphingobium_uncultured anaerobic bacterium	Proteobacteria	6.82977318	3.57E-06
	ASV10_Cyanobacteria_Phaseolus acutifolius	Cyanobacteria	2.87457492	4.17E-06

	ASV99_Bituminaria bituminosa	Proteobacteria	2.81074955	0.00026816
	ASV710_Cellvibrio_uncultured bacterium	Proteobacteria	5.88457785	0.00120863
	ASV303_Chitinophaga sp. S136	Bacteroidetes	7.90479644	0.00262064
	ASV756_Noviherbaspirillum_uncultured beta proteobacterium	Proteobacteria	5.86169994	0.00262064
	ASV776_Chitinophagales_uncultured bacterium	Bacteroidetes	5.33144631	0.00262064
	ASV24_Cyanobacteria_Citrus maxima	Cyanobacteria	2.39766567	0.00262064
	ASV810_Burkholderiaceae_metagenome	Proteobacteria	4.68412958	0.00285428
Setm4 vs UT	ASV307_Erythrobacter sp. CJ012	Proteobacteria	7.98215488	0.00087425
	ASV358_Flavobacterium	Bacteroidetes	7.91931192	0.00013589
	ASV233_Fluviicola hefeinensis	Bacteroidetes	7.41964903	2.16E-09
	ASV628_Achromobacter xylosidans	Proteobacteria	6.64346791	1.00E-06
	ASV609_Sporomusa rhizae	Firmicutes	6.46405784	0.00054258
	ASV631_Thermobrachium_uncultured bacterium	Firmicutes	6.37712277	0.00063335
	ASV640_uncultured Xanthomonadaceae	Proteobacteria	6.15572446	0.000985
	ASV454_Flavobacterium_bacterium ST60	Bacteroidetes	7.21747021	0.00211374
	ASV503_Sphingomonadales_uncultured bacterium	Proteobacteria	7.03596069	0.00707509
	ASV705_Cellvibrio diazotrophicus	Proteobacteria	6.14308474	0.03168061

	Depleted ASVs at the species level	Phylum	Log2 FC	P-adjusted value
Set2 vs UT	ASV98_uncultured Chryseobacterium sp.	Bacteroidetes	-10.39095	0.00109796
	ASV249_uncultured beta proteobacterium	Proteobacteria	-7.6490527	5.98E-13
	ASV614_Calothrix_uncultured bacterium	Cyanobacteria	-6.9031964	0.00262064
	ASV537_Clostridium sensu stricto 1	Firmicutes	-6.7288043	4.33E-06

	ASV158_Sphingobacterium sp.	Bacteroidetes	-6.7099462	0.06254051
	ASV271_Taibaiella koreensis	Bacteroidetes	-6.6482348	0.02154621
	ASV610_uncultured Chloroflexi bacterium	Chloroflexi	-6.5784567	0.02140015
	ASV505_uncultured Bradyrhizobium sp.	Proteobacteria	-6.4830762	1.32E-05
	ASV42_Sphingobacteriaceae	Bacteroidetes	-6.4256728	0.03748567
	ASV728_Holophagaceae_uncultured bacterium	Acidobacteria	-6.3104212	0.02798081
Setm4 vs UT	ASV98_uncultured Chryseobacterium sp.	Bacteroidetes	-10.362983	0.00087425
	ASV158_Sphingobacterium sp.	Bacteroidetes	-9.6093275	0.00161667
	ASV414_Phormidium uncinatum SAG 81.79	Cyanobacteria	-7.3967855	0.0122207
	ASV614_Calothrix_uncultured bacterium	Cyanobacteria	-6.8541258	0.00013589
	ASV610_uncultured Chloroflexi bacterium	Chloroflexi	-6.5258419	0.02167682
	ASV728_Holophagaceae_uncultured bacterium	Acidobacteria	-6.2578852	0.02767887
	ASV612_Noviherbaspirillum_uncultured bacterium	Proteobacteria	-5.8964311	0.00193809
	ASV823_Kineosporiales_uncultured bacterium	Actinobacteria	-5.6138394	0.00088374
	ASV982_Paenibacillus sp. KJ006	Firmicutes	-5.3258517	0.02167682
	ASV917_uncultured Dokdonella sp.	Proteobacteria	-5.0996666	0.0122207

Table S7. Co-occurrence of the top abundant species in each treatment

Co-occurrence pattern between species in the root endosphere			
Nodes affiliation N1-N2	Random[1]	Observed[2]	O/R ratio[3]
**Set2**			
Agrobacterium radiobacter_uncultured Phenylobacterium sp.	0.150829563	0.354609929	2.35106383
Agrobacterium radiobacter uncultured bacterium	10.93514329	0.354609929	0.032428467
Enterobacter hormaechei subsp. hormaechei__Bituminaria bituminosa	0.075414781	0.354609929	4.70212766
Enterobacter hormaechei subsp. hormaechei__Citrus maxima	0.075414781	0.354609929	4.70212766
Enterobacter hormaechei subsp. hormaechei__Cyanobacteria_Phaseolus acutifolius (tepary bean)	0.075414781	0.354609929	4.70212766
Pseudomonas sp. D4__Agrobacterium radiobacter	0.075414781	0.354609929	4.70212766
Pseudomonas sp. D4__Dyadobacter	0.075414781	0.354609929	4.70212766
Pseudomonas sp. D4__Rhizobium sp. CG37	0.075414781	0.354609929	4.70212766
**Setm4**			
Achromobacter xylosoxidans subsp. xylosoxidans__Bradyrhizobium elkanii	0.075414781	0.354609929	4.70212766
Achromobacter xylosoxidans subsp. xylosoxidans__bacterium ST1(2015)	0.075414781	0.354609929	4.70212766
Achromobacter xylosoxidans subsp. _Xylosoxidans__Allorhizobium-Neorhizobium-Pararhizobium-Rhizobium	0.075414781	0.354609929	4.70212766
Enterobacter hormaechei subsp. hormaechei__Bituminaria bituminosa	0.075414781	0.354609929	4.70212766
Enterobacter hormaechei subsp. hormaechei__Citrus maxima	0.075414781	0.354609929	4.70212766
Enterobacter hormaechei subsp. hormaechei__Cyanobacteria_Phaseolus acutifolius (tepary bean)	0.075414781	0.354609929	4.70212766

Co-occurrence pattern between species in the root endosphere			
Nodes affiliation N1-N2	Random[1]	Observed[2]	O/R ratio[3]
Pseudomonas sp. D4__Agrobacterium radiobacter	0.075414781	0.354609929	4.70212766
Pseudomonas sp. D4__Dyadobacter	0.075414781	0.354609929	4.70212766
Pseudomonas sp. D4__Rhizobium sp. CG37	0.075414781	0.354609929	4.70212766
Pseudomonas sp. D4__Sphingobacterium sp.	0.075414781	0.354609929	4.70212766
**Co-occurrence pattern between species in the rhizosphere**			
**Set2**			
Agrobacterium radiobacter__Acidibacter	0.000529647	0.003035178	5.730567275
Agrobacterium radiobacter__Amycolatopsis_uncultured bacterium	0.000529647	0.003035178	5.730567275
Agrobacterium radiobacter__Candidatus Udaeobacter	0.001059294	0.006070355	5.730567275
Agrobacterium radiobacter__Chitinophaga_ambiguous	0.000529647	0.003035178	5.730567275
Agrobacterium radiobacter__Chitinophagaceae	0.000529647	0.003035178	5.730567275
Agrobacterium radiobacter__Chitinophagaceae_uncultured bacterium	0.000529647	0.003035178	5.730567275
Agrobacterium radiobacter__Cupriavidus sp. UYMMaPT13	0.000529647	0.003035178	5.730567275
Agrobacterium radiobacter__Flavisolibacter	0.001059294	0.003035178	2.865283637
Agrobacterium radiobacter__Mucilaginibacter_uncultured bacterium	0.000529647	0.003035178	5.730567275
Agrobacterium radiobacter__Pseudonocardia sp. ARG1	0.000529647	0.003035178	5.730567275
Agrobacterium radiobacter__Solibacteraceae (Subgroup 3)	0.000529647	0.003035178	5.730567275
Agrobacterium radiobacter__Sphingomonas_uncultured bacterium	0.000529647	0.003035178	5.730567275
Agrobacterium radiobacter__Streptomyces sp. P1403U	0.000529647	0.003035178	5.730567275
Agrobacterium radiobacter__WD2101 soil group_uncultured bacterium	0.000529647	0.003035178	5.730567275

Co-occurrence pattern between species in the root endosphere			
Nodes affiliation N1-N2	Random[1]	Observed[2]	O/R ratio[3]
Agrobacterium radiobacter__eubacterium sp. 11-14	0.000529647	0.003035178	5.730567275
Agrobacterium radiobacter__uncultured Chitinophagaceae bacterium	0.001059294	0.003035178	2.865283637
Agrobacterium radiobacter__uncultured Sphingomonas sp.	0.000529647	0.003035178	5.730567275
Agrobacterium radiobacter__uncultured bacterium	0.306665607	0.14568853	0.475072935
Azotobacter sp.__Altererythrobacter	0.001059294	0.003035178	2.865283637
Azotobacter sp.__Betaproteobacteriales	0.000529647	0.003035178	5.730567275
Azotobacter sp.__Chryseobacterium	0.000529647	0.003035178	5.730567275
Azotobacter sp.__Chthonomonadales	0.000529647	0.003035178	5.730567275
Azotobacter sp.__Flavisolibacter_uncultured Bacteroidetes bacterium	0.000529647	0.003035178	5.730567275
Azotobacter sp.__Gaiellales_uncultured bacterium	0.000529647	0.003035178	5.730567275
Azotobacter sp.__Geobacter	0.001059294	0.003035178	2.865283637
Azotobacter sp.__Rhizobiales	0.002118588	0.003035178	1.432641819
Azotobacter sp.__Rhodanobacter	0.000529647	0.003035178	5.730567275
Azotobacter sp.__SBR1031_uncultured bacterium	0.001059294	0.003035178	2.865283637
Azotobacter sp.__Sandaracinaceae_uncultured bacterium	0.000529647	0.003035178	5.730567275
Azotobacter sp.__bacterium Ellin501	0.000529647	0.003035178	5.730567275
Azotobacter sp.__uncultured Acidobacteriaceae bacterium	0.003177882	0.003035178	0.955094546
Azotobacter sp.__uncultured Chitinophagaceae bacterium	0.001059294	0.003035178	2.865283637
Azotobacter sp.__uncultured Ktedobacteria bacterium	0.002648235	0.003035178	1.146113455
Azotobacter sp.__uncultured Phenylobacterium sp.	0.001059294	0.003035178	2.865283637

Co-occurrence pattern between species in the root endosphere			
Nodes affiliation N1-N2	Random[1]	Observed[2]	O/R ratio[3]
Azotobacter sp.__uncultured bacterium	0.306665607	0.121407108	0.395894112
Bacillus sp.__Amycolatopsis_uncultured bacterium	0.001059294	0.003035178	2.865283637
Bacillus sp.__Candidatus Udaeobacter_uncultured bacterium	0.001059294	0.003035178	2.865283637
Bacillus sp.__Chitinophaga_ambiguous	0.001059294	0.006070355	5.730567275
Bacillus sp.__Chitinophaga_uncultured bacterium	0.001059294	0.003035178	2.865283637
Bacillus sp.__Chitinophagaceae	0.001059294	0.006070355	5.730567275
Bacillus sp.__Chitinophagaceae_uncultured bacterium	0.001059294	0.006070355	5.730567275
Bacillus sp.__Dyadobacter	0.001059294	0.003035178	2.865283637
Bacillus sp.__Fluviicola_uncultured bacterium	0.001059294	0.003035178	2.865283637
Bacillus sp.__Mucilaginibacter_uncultured bacterium	0.001059294	0.003035178	2.865283637
Bacillus sp.__Niastella_uncultured bacterium	0.001059294	0.003035178	2.865283637
Bacillus sp.__Novosphingobium_uncultured bacterium	0.001059294	0.003035178	2.865283637
Bacillus sp.__Pyrinomonadaceae_uncultured bacterium	0.001059294	0.003035178	2.865283637
Bacillus sp.__Roseiflexaceae	0.002118588	0.003035178	1.432641819
Bacillus sp.__SBR1031_uncultured bacterium	0.002118588	0.003035178	1.432641819
Bacillus sp.__Sphingomonas_uncultured bacterium	0.001059294	0.006070355	5.730567275
Bacillus sp.__Streptomyces sp. P1403U	0.001059294	0.006070355	5.730567275
Bacillus sp.__eubacterium sp. 11-14	0.001059294	0.006070355	5.730567275
Enterobacter hormaechei subsp. hormaechei__Acidobacteriales_uncultured bacterium	0.000529647	0.003035178	5.730567275
Enterobacter hormaechei subsp. hormaechei__Bradyrhizobium elkanii	0.000529647	0.003035178	5.730567275
Enterobacter hormaechei subsp. hormaechei__Candidatus Solibacter_uncultured bacterium	0.000529647	0.003035178	5.730567275

Co-occurrence pattern between species in the root endosphere			
Nodes affiliation N1-N2	Random[1]	Observed[2]	O/R ratio[3]
Enterobacter hormaechei subsp. hormaechei__Cyanobacteria_Citrus maxima	0.000529647	0.003035178	5.730567275
Enterobacter hormaechei subsp. hormaechei__Pedosphaeraceae_uncultured bacterium	0.000529647	0.003035178	5.730567275
Streptomyces sp. ___Acidibacter	0.000529647	0.003035178	5.730567275
Streptomyces sp. ___Acidobacteria bacterium AB60	0.000529647	0.003035178	5.730567275
Streptomyces sp. ___Actinosynnema mirum	0.000529647	0.003035178	5.730567275
Streptomyces sp. ___Agrobacterium radiobacter	0.000529647	0.003035178	5.730567275
Streptomyces sp. ___Allorhizobium-Neorhizobium-Pararhizobium-Rhizobium	0.000529647	0.003035178	5.730567275
Streptomyces sp. ___Bacillus sp.	0.001059294	0.006070355	5.730567275
Streptomyces sp. ___Bacillus sp. P-1(2016)	0.000529647	0.003035178	5.730567275
Streptomyces sp. ___Bradyrhizobium sp. 124L2DEPT	0.000529647	0.003035178	5.730567275
Streptomyces sp. ___Candidatus Udaeobacter_uncultured bacterium	0.000529647	0.003035178	5.730567275
Streptomyces sp. ___Chitinophaga japonensis	0.000529647	0.003035178	5.730567275
Streptomyces sp. ___Chitinophaga_ambiguous	0.000529647	0.003035178	5.730567275
Streptomyces sp. ___Chitinophaga_uncultured bacterium	0.000529647	0.003035178	5.730567275
Streptomyces sp. ___Chitinophagaceae	0.000529647	0.003035178	5.730567275
Streptomyces sp. ___Chitinophagaceae_uncultured bacterium	0.000529647	0.003035178	5.730567275
Streptomyces sp. ___Chthoniobacter	0.000529647	0.003035178	5.730567275
Streptomyces sp. ___Comamonas	0.000529647	0.003035178	5.730567275
Streptomyces sp. ___Cupriavidus sp. UYMMaPT13	0.000529647	0.003035178	5.730567275
Streptomyces sp. ___Dongia	0.001059294	0.003035178	2.865283637

Co-occurrence pattern between species in the root endosphere			
Nodes affiliation N1-N2	Random[1]	Observed[2]	O/R ratio[3]
Streptomyces sp. ___Dyella sp. FP0	0.000529647	0.003035178	5.730567275
Streptomyces sp. ___Flavisolibacter	0.001059294	0.006070355	5.730567275
Streptomyces sp. ___Gemmatimonas	0.001059294	0.003035178	2.865283637
Streptomyces sp. ___Janthinobacterium sp. S139	0.000529647	0.003035178	5.730567275
Streptomyces sp. ___Lysobacter sp. Z2-YC6852	0.000529647	0.003035178	5.730567275
Streptomyces sp. ___Mesorhizobium sp. T20	0.000529647	0.003035178	5.730567275
Streptomyces sp. ___Mycobacterium	0.000529647	0.003035178	5.730567275
Streptomyces sp. ___Mycobacterium sp.	0.000529647	0.003035178	5.730567275
Streptomyces sp. ___Opitutus	0.001059294	0.003035178	2.865283637
Streptomyces sp. ___Pseudonocardia sp. ARG1	0.000529647	0.003035178	5.730567275
Streptomyces sp. ___Pseudoxanthomonas	0.000529647	0.003035178	5.730567275
Streptomyces sp. ___Ralstonia	0.000529647	0.003035178	5.730567275
Streptomyces sp. ___Rhizobium sp. CG37	0.000529647	0.003035178	5.730567275
Streptomyces sp. ___Rhizobium sp. rf052	0.000529647	0.003035178	5.730567275
Streptomyces sp. ___Roseiflexaceae	0.001059294	0.003035178	2.865283637
Streptomyces sp. ___Sphingomonas jaspsi	0.000529647	0.003035178	5.730567275
Streptomyces sp. ___Sphingomonas_uncultured bacterium	0.000529647	0.003035178	5.730567275
Streptomyces sp. ___Streptomyces sp.	0.001059294	0.003035178	2.865283637
Streptomyces sp. ___Streptomyces sp. NCCP-1200	0.000529647	0.003035178	5.730567275
Streptomyces sp. ___Streptomyces sp. P1403U	0.000529647	0.003035178	5.730567275
Streptomyces sp. ___Taibaiella soli	0.000529647	0.003035178	5.730567275
Streptomyces sp. ___Tetradesmus obliquus	0.000529647	0.003035178	5.730567275
Streptomyces sp. ___eubacterium sp. 11-14	0.000529647	0.003035178	5.730567275

Co-occurrence pattern between species in the root endosphere			
Nodes affiliation N1-N2	Random[1]	Observed[2]	O/R ratio[3]
Streptomyces sp. ___uncultured Caulobacter sp.	0.001059294	0.003035178	2.865283637
Streptomyces sp. ___uncultured Chitinophagaceae bacterium	0.001059294	0.003035178	2.865283637
Streptomyces sp. ___uncultured Comamonadaceae bacterium	0.001588941	0.003035178	1.910189092
Streptomyces sp. ___uncultured Novosphingobium sp.	0.000529647	0.003035178	5.730567275
Streptomyces sp. ___uncultured Sphingomonadaceae bacterium	0.000529647	0.003035178	5.730567275
Streptomyces sp. ___uncultured Terrimonas sp.	0.000529647	0.003035178	5.730567275
**Setm4**			
Achromobacter xylosoxidans subsp. xylosoxidans__Bradyrhizobium elkanii	0.075414781	0.255754476	3.391304348
Achromobacter xylosoxidans subsp. xylosoxidans__Burkholderia-Caballeronia-Paraburkholderia	0.000614496	0.002662478	4.332783088
Achromobacter xylosoxidans subsp. xylosoxidans__Chitinophagaceae	0.075414781	0.255754476	3.391304348
Achromobacter xylosoxidans subsp. xylosoxidans__Chryseobacterium	0.075414781	0.255754476	3.391304348
Achromobacter xylosoxidans subsp. xylosoxidans__Cupriavidus sp. UYMMaPT13	0.000614496	0.002662478	4.332783088
Achromobacter xylosoxidans subsp. xylosoxidans__Dyadobacter	0.075414781	0.255754476	3.391304348
Achromobacter xylosoxidans subsp. xylosoxidans__Gemmatimonadaceae	0.000614496	0.002662478	4.332783088
Achromobacter xylosoxidans subsp. xylosoxidans__Lysobacter	0.000614496	0.002662478	4.332783088
Achromobacter xylosoxidans subsp. xylosoxidans__MM1	0.000614496	0.002662478	4.332783088

Co-occurrence pattern between species in the root endosphere			
Nodes affiliation N1-N2	Random[1]	Observed[2]	O/R ratio[3]
Achromobacter xylosoxidans subsp. xylosoxidans__Mucilaginibacter_uncultured bacterium	0.075414781	0.255754476	3.391304348
Agrobacterium radiobacter__Amycolatopsis_uncultured bacterium	0.075414781	0.255754476	3.391304348
Agrobacterium radiobacter__Candidatus Koribacter	0.075414781	0.255754476	3.391304348
Agrobacterium radiobacter__Candidatus Udaeobacter_uncultured bacterium	0.075414781	0.255754476	3.391304348
Agrobacterium radiobacter__Cyanobacteria_Citrus maxima	0.075414781	0.255754476	3.391304348
Agrobacterium radiobacter__Micropepsaceae_uncultured	0.075414781	0.255754476	3.391304348
Agrobacterium radiobacter__Niastella_uncultured bacterium	0.075414781	0.255754476	3.391304348
Agrobacterium radiobacter__Pedosphaeraceae	0.075414781	0.255754476	3.391304348
Agrobacterium radiobacter__Pseudomonas sp. D4	0.075414781	0.255754476	3.391304348
Agrobacterium radiobacter__Sandaracinaceae_uncultured bacterium	0.075414781	0.255754476	3.391304348
Agrobacterium radiobacter__Solibacteraceae (Subgroup 3)	0.075414781	0.255754476	3.391304348
Agrobacterium radiobacter__Sphingomonas_uncultured bacterium	0.075414781	0.255754476	3.391304348
Agrobacterium radiobacter__WD2101 soil group_uncultured bacterium	0.075414781	0.255754476	3.391304348
Agrobacterium radiobacter__uncultured Phenylobacterium sp.	0.075414781	0.255754476	3.391304348
Bacillus sp.__Acidobacteriales_uncultured bacterium	0.150829563	0.255754476	1.695652174
Bacillus sp.__Amycolatopsis_uncultured bacterium	0.150829563	0.255754476	1.695652174
Bacillus sp.__Burkholderiaceae	0.150829563	0.255754476	1.695652174
Bacillus sp.__Chitinophagaceae_uncultured bacterium	0.150829563	0.255754476	1.695652174

Co-occurrence pattern between species in the root endosphere			
Nodes affiliation N1-N2	Random[1]	Observed[2]	O/R ratio[3]
Bacillus sp.__Gaiellales_uncultured bacterium	0.150829563	0.255754476	1.695652174
Bacillus sp.__Gemmatimonadaceae_uncultured bacterium	0.150829563	0.255754476	1.695652174
Bacillus sp.__Microcoleus Es-Yyy1400_uncultured microorganism	0.150829563	0.255754476	1.695652174
Bacillus sp.__Microscillaceae_uncultured bacterium	0.150829563	0.255754476	1.695652174
Bacillus sp.__Myxococcales_uncultured bacterium	0.150829563	0.255754476	1.695652174
Bacillus sp.__Pedosphaeraceae_uncultured bacterium	0.150829563	0.255754476	1.695652174
Bacillus sp.__Pyrinomonadaceae_uncultured bacterium	0.150829563	0.255754476	1.695652174
Bacillus sp.__Roseiflexaceae	0.150829563	0.255754476	1.695652174
Bacillus sp.__SBR1031_uncultured bacterium	0.301659125	0.511508951	1.695652174
Bacillus sp.__Sphingomonas_uncultured bacterium	0.150829563	0.255754476	1.695652174
Bacillus sp.__uncultured Acidobacteria bacterium	0.301659125	0.511508951	1.695652174
Bacillus sp.__uncultured bacterium	0.452488688	0.511508951	1.130434783
Bacillus thuringiensis__Acidobacteriales_uncultured bacterium	0.075414781	0.255754476	3.391304348
Bacillus thuringiensis__Anaerolineaceae_uncultured bacterium	0.075414781	0.255754476	3.391304348
Bacillus thuringiensis__Bacillus sp.	0.150829563	0.255754476	1.695652174
Bacillus thuringiensis__Chitinophaga_uncultured bacterium	0.075414781	0.255754476	3.391304348
Bacillus thuringiensis__Chitinophagaceae_uncultured bacterium	0.075414781	0.255754476	3.391304348
Bacillus thuringiensis__Gaiellales_uncultured bacterium	0.075414781	0.255754476	3.391304348
Bacillus thuringiensis__Gemmatimonadaceae_uncultured bacterium	0.075414781	0.255754476	3.391304348

Co-occurrence pattern between species in the root endosphere			
Nodes affiliation N1-N2	Random[1]	Observed[2]	O/R ratio[3]
Bacillus thuringiensis__Georgfuchsia_uncultured bacterium	0.075414781	0.255754476	3.391304348
Bacillus thuringiensis__Microcoleus Es-Yyy1400_uncultured microorganism	0.075414781	0.255754476	3.391304348
Bacillus thuringiensis__Microscillaceae_uncultured bacterium	0.075414781	0.255754476	3.391304348
Bacillus thuringiensis__Myxococcales_uncultured bacterium	0.075414781	0.255754476	3.391304348
Bacillus thuringiensis__Pedosphaeraceae_uncultured bacterium	0.075414781	0.255754476	3.391304348
Bacillus thuringiensis__Pyrinomonadaceae_uncultured bacterium	0.075414781	0.255754476	3.391304348
Bacillus thuringiensis__Roseiflexaceae	0.075414781	0.255754476	3.391304348
Bacillus thuringiensis__SBR1031_uncultured bacterium	0.150829563	0.511508951	3.391304348
Bacillus thuringiensis__Streptomyces sp. P1403U	0.075414781	0.255754476	3.391304348
Bacillus thuringiensis__eubacterium sp. 11-14	0.075414781	0.255754476	3.391304348
Bacillus thuringiensis__uncultured Acidobacteria bacterium	0.150829563	0.255754476	1.695652174
Bacillus thuringiensis__uncultured Verrucomicrobia bacterium	0.075414781	0.255754476	3.391304348
Bacillus thuringiensis__uncultured bacterium	0.226244344	0.511508951	2.260869565
Bradyrhizobium elkanii__Novosphingobium_uncultured bacterium	0.075414781	0.255754476	3.391304348
Pseudomonas sp. D4__Amycolatopsis_uncultured bacterium	0.075414781	0.255754476	3.391304348
Pseudomonas sp. D4__Bacillus sp.	0.150829563	0.255754476	1.695652174
Pseudomonas sp. D4__Candidatus Koribacter	0.075414781	0.255754476	3.391304348
Pseudomonas sp. D4__Candidatus Solibacter_uncultured bacterium	0.075414781	0.255754476	3.391304348

Co-occurrence pattern between species in the root endosphere			
Nodes affiliation N1-N2	Random[1]	Observed[2]	O/R ratio[3]
Pseudomonas sp. D4__Candidatus Udaeobacter_uncultured bacterium	0.075414781	0.255754476	3.391304348
Pseudomonas sp. D4__Chitinophaga_uncultured bacterium	0.075414781	0.255754476	3.391304348
Pseudomonas sp. D4__Cyanobacteria_Citrus maxima	0.075414781	0.255754476	3.391304348
Pseudomonas sp. D4__Cyanobacteria_Phaseolus acutifolius (tepary bean)	0.075414781	0.255754476	3.391304348
Pseudomonas sp. D4__Flavisolibacter_uncultured Bacteroidetes bacterium	0.075414781	0.255754476	3.391304348
Pseudomonas sp. D4__Niastella_uncultured bacterium	0.075414781	0.255754476	3.391304348
Pseudomonas sp. D4__Pedosphaeraceae	0.075414781	0.255754476	3.391304348
Pseudomonas sp. D4__Sandaracinaceae_uncultured bacterium	0.075414781	0.255754476	3.391304348
Pseudomonas sp. D4__Solibacteraceae (Subgroup 3)	0.075414781	0.255754476	3.391304348
Pseudomonas sp. D4__Sphingomonas_uncultured bacterium	0.075414781	0.255754476	3.391304348
Pseudomonas sp. D4__uncultured Acidobacteria bacterium	0.150829563	0.255754476	1.695652174
Pseudomonas sp. D4__uncultured Phenylobacterium sp.	0.075414781	0.255754476	3.391304348
Pseudomonas sp. D4__uncultured Verrucomicrobia bacterium	0.075414781	0.255754476	3.391304348
Streptomyces sp. ___Acidobacteriales_ambiguous	0.000614496	0.002662478	4.332783088
Streptomyces sp. ___Amycolatopsis_uncultured bacterium	0.000614496	0.002662478	4.332783088
Streptomyces sp. ___Azotobacter sp.	0.000614496	0.002662478	4.332783088
Streptomyces sp. ___Bacillus sp.	0.001228992	0.002662478	2.166391544
Streptomyces sp. ___Betaproteobacteriales	0.000614496	0.002662478	4.332783088
Streptomyces sp. ___Bituminaria bituminosa	0.000614496	0.002662478	4.332783088

Co-occurrence pattern between species in the root endosphere			
Nodes affiliation<br>N1-N2	Random[1]	Observed[2]	O/R ratio[3]
Streptomyces sp. ___Candidatus Solibacter_uncultured bacterium	0.000614496	0.002662478	4.332783088
Streptomyces sp. ___Candidatus Udaeobacter	0.001228992	0.002662478	2.166391544
Streptomyces sp. ___Candidatus Udaeobacter_uncultured bacterium	0.000614496	0.002662478	4.332783088
Streptomyces sp. ___Cyanobacteria_Citrus maxima	0.000614496	0.002662478	4.332783088
Streptomyces sp. ___Cyanobacteria_Phaseolus acutifolius (tepary bean)	0.000614496	0.002662478	4.332783088
Streptomyces sp. ___Cyanobacteriua_ambiguous	0.000614496	0.002662478	4.332783088
Streptomyces sp. ___Flavisolibacter_uncultured Bacteroidetes bacterium	0.000614496	0.002662478	4.332783088
Streptomyces sp. ___Mycobacterium	0.000614496	0.002662478	4.332783088
Streptomyces sp. ___Niastella_uncultured bacterium	0.000614496	0.002662478	4.332783088
Streptomyces sp. ___Pedosphaeraceae	0.001228992	0.002662478	2.166391544
Streptomyces sp. ___Persicaria minor	0.001228992	0.002662478	2.166391544
Streptomyces sp. ___Pseudomonas sp. D4	0.000614496	0.002662478	4.332783088
Streptomyces sp. ___Rhizobium sp. CG37	0.000614496	0.002662478	4.332783088
Streptomyces sp. ___Sandaracinaceae_uncultured bacterium	0.000614496	0.002662478	4.332783088
Streptomyces sp. ___Solibacteraceae (Subgroup 3)	0.000614496	0.002662478	4.332783088
Streptomyces sp. ___Sphingomonas_uncultured bacterium	0.000614496	0.002662478	4.332783088
Streptomyces sp. ___Streptomyces chartreusis	0.000614496	0.002662478	4.332783088
Streptomyces sp. ___Streptomyces sp. NCCP-1200	0.000614496	0.002662478	4.332783088
Streptomyces sp. ___metagenome	0.082956955	0.010649911	0.128378758
Streptomyces sp. ___uncultured Acidobacteria bacterium	0.012904415	0.005324955	0.412646008

Co-occurrence pattern between species in the root endosphere

Nodes affiliation N1-N2	Random[1]	Observed[2]	O/R ratio[3]
Streptomyces sp. ___uncultured Acidobacteriales bacterium	0.004915968	0.005324955	1.083195772
Streptomyces sp. ___uncultured Comamonadaceae bacterium	0.001843488	0.002662478	1.444261029
Streptomyces sp. ___uncultured Gemmatimonadetes bacterium	0.001843488	0.002662478	1.444261029
Streptomyces sp. ___uncultured Ktedobacteria bacterium	0.00307248	0.005324955	1.733113235
Streptomyces sp. ___uncultured Phenylobacterium sp.	0.001228992	0.002662478	2.166391544
Streptomyces sp. ___uncultured bacterium	0.355793161	0.133123885	0.374160888
Streptomyces sp. ___uncultured crenarchaeote	0.001843488	0.002662478	1.444261029
Streptomyces sp. ___uncultured soil bacterium	0.040556733	0.013312389	0.328241143

[1] Random co-occurring incidence (R) is the theoretical incidence of co-occurrence calculated by considering the taxa frequencies and assuming random association between them

[2] Observed co-occurring incidence (O) of two taxa was calculated as the relative percentage of the number of observed edges between them in the total edges in the network.

3 O/R ratio is the degree of the lack of agreement between O and R as a benchmark for checking nonrandom assembly patterns in complex bacterial communities.

Table S8. Network topological characteristics of microbial communities with corresponding statistics calculated based on co-occurrence network comprising all significant microbe-microbe interactions at α < 0.01

Microbial network	Nodes (OTUs)	Edges	Average Clustering Coefficient (ACC)	Average Path Length (APL)	Diameter	Modularity/ Modules
Endosphere						
Untreated	52	282	0.656	3.04	6	(0.484) 5
Set2	52	282	0.656	3.04	6	(0.430) 5
Setm4	52	282	0.656	3.04	6	(0.484) 5
Rhizosphere						
Untreated	507	31556	0.662	2.747	6	(0.365) 4
Set2	615	32947	0.687	2.828	6	(0.539) 5
Setm4	571	37559	0.719	2.864	7	(0.476) 4

*ACC-Average Clustering Coefficient: a measure of the degree to which nodes in a graph tend to cluster

APL-Average Path Length: mean of the lengths of the shortest paths between all pairs of nodes in the network

Diameter: the longest path length between any pair of nodes

Modularity: a measure of the structure of networks or graphs which measures the strength of division of a network into modules.

3 Supplementary Figures

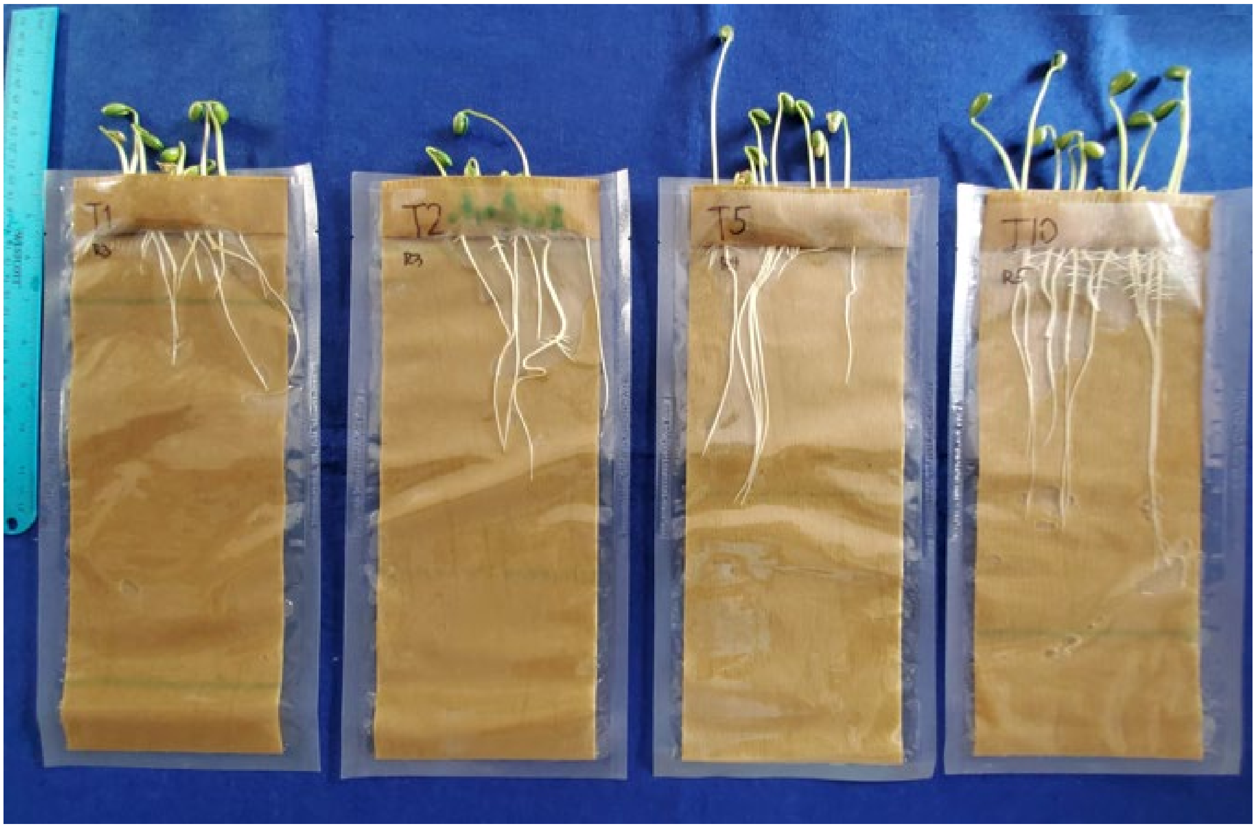

Figure S1. A representative image of the seedling growth assay in the laboratory condition using plastic pouches. ‘Comm FC’ means a commercial fungicide. The photo shows the 5-day-old seedlings in this study.

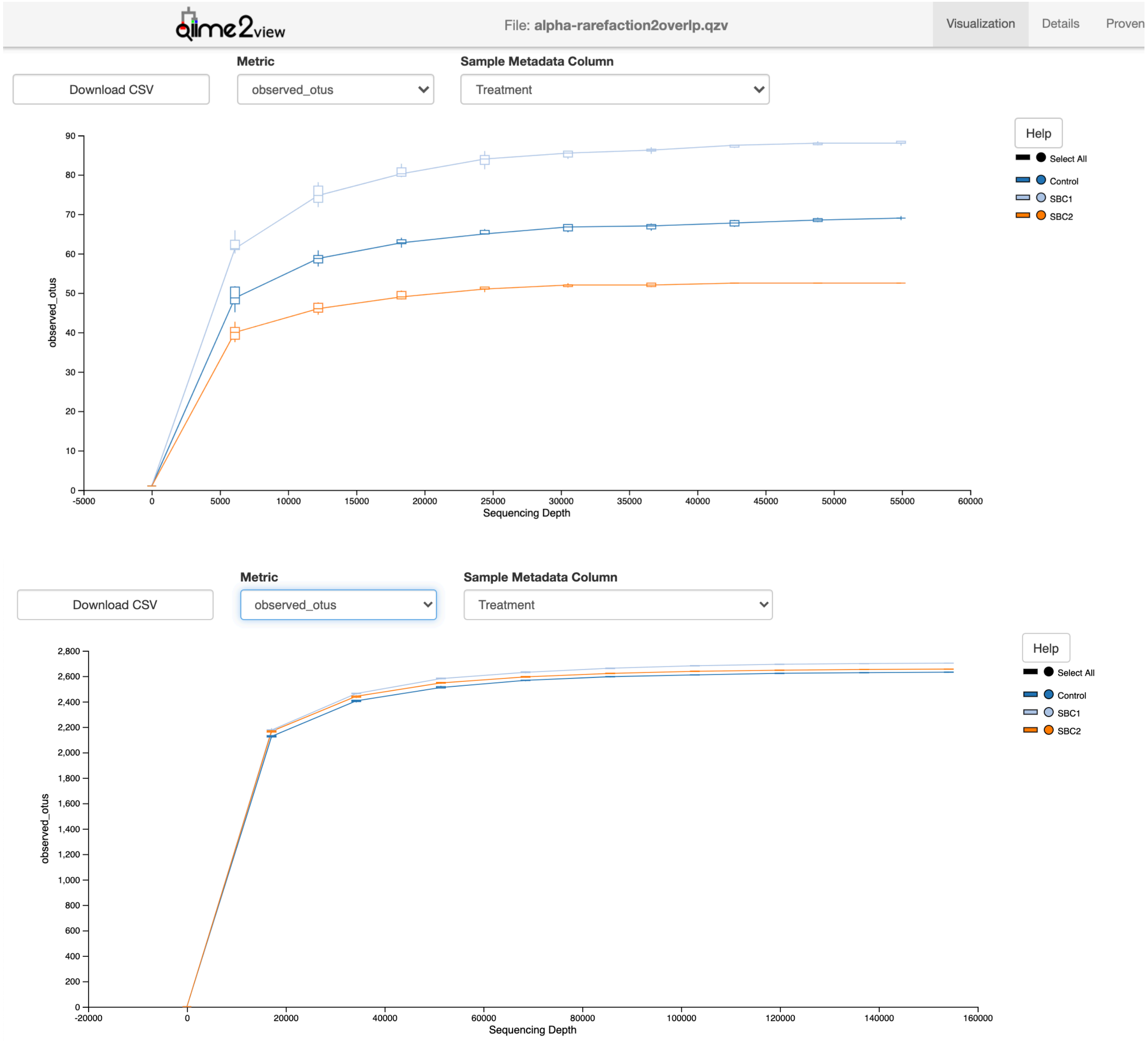

Figure S2. The alpha rarefaction curves based on observed features which showed sufficient proportion of diversity represented in the microbiome sampling.

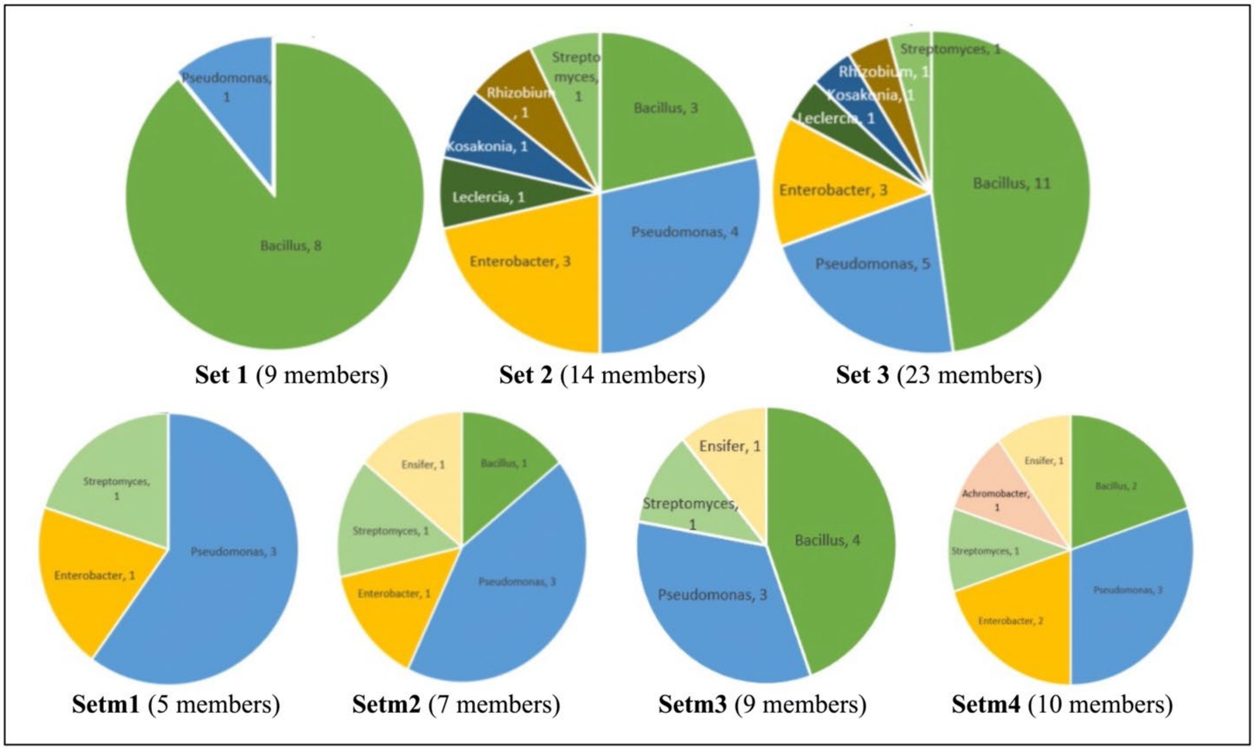

Figure S3. Pie charts showing the composition of the seven SABB consortia and minimal subsets. Each section represents the number of isolates based on their genus classification. The SABB consortia members were formulated based on the *in vitro* co culture compatibility assay.

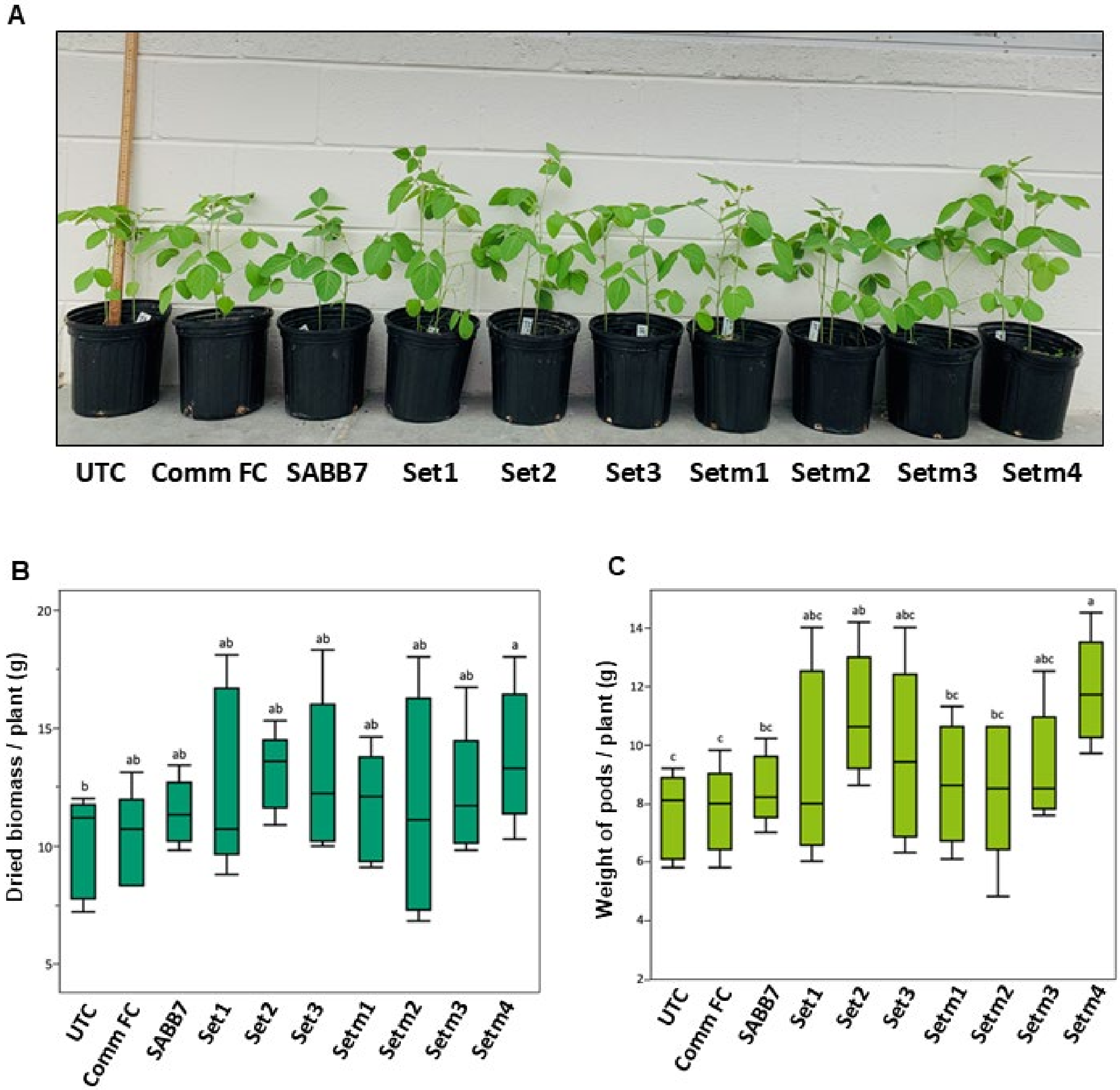

Figure S4. Growth of soybean plants, dry weight plant biomass, and weight of pods per plant in a greenhouse condition with seed that was either untreated, treated with SABB7, or with SABB consortia and minimal subsets. (A) Representative soybean plants of the different treatments at 32 days after planting. (B) Boxplot of the dry weight plant biomass per plant at harvest. (C) Dry weight of pods per plant at harvest. Error bars indicate standard error of the mean from three replications. Bars with a common letter are not significantly different based on LSD tests at 5% probability level.

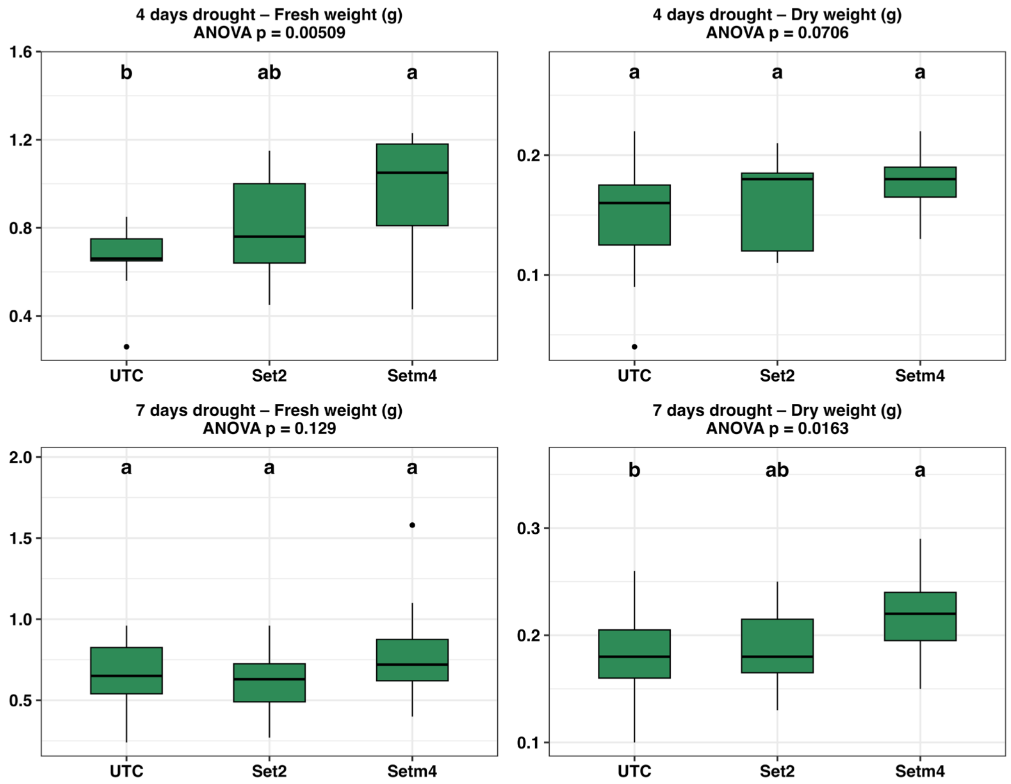

Figure S5. Effects of synthetic bacterial community (SBC) treatments on soybean seedling growth in terms of biomass under drought stress (n=15 replications).

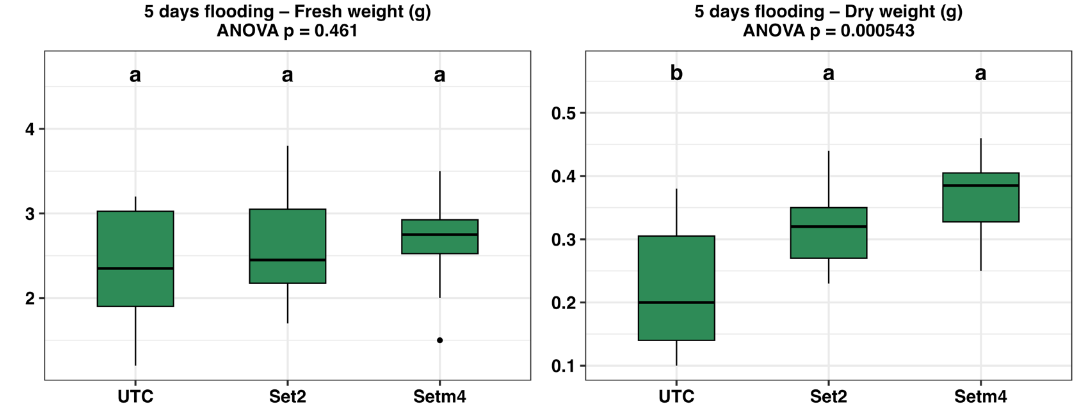

Figure S6. Effects of synthetic bacterial community (SBC) treatments on soybean seedling growth in terms of biomass under water-logging stress (n=12 replications).

References

Alexander, D. B., and D. A. Zuberer. 1991. "Use of chrome azurol S reagents to evaluate siderophore production by rhizosphere bacteria." *Biology and Fertility of Soils* 12 (1): 39-45. https://doi.org/https://doi.org/10.1007/BF00369386.

Gordon, S. A., and R. P. Weber. 1951. "Colorimetric estimation of indoleacetic acid." *Plant Physiology* 26 (1): 192-195. https://doi.org/10.1104/pp.26.1.192.

Jensen, H. L. 1942. "Nitrogen fixation by non-symbiotic bacteria." *Proceedings of the Linnean Society of New South Wales* 57: 205-212.

Pikovskaya, R. I. 1948. "Mobilization of phosphorous in soil in connection with the vital activity of some microbial species." *Microbiologiya* 17: 362-370.

Shrestha, B. K., H. S. Karki, D. E. Groth, N. Jungkhun, and J. H. Ham. 2016. "Biological control activities of rice-associated *Bacillus* sp. strains against sheath blight and bacterial panicle blight of rice." *PLoS One* 11 (1): e0146764. https://doi.org/10.1371/journal.pone.0146764.

Shruti, K., K. Arun, and R. Yuvnee. 2013. "Potential plant growth-promoting activity of rhizobacteria *Pseudomonas* sp. in *Oryza sativa*." *Journal of National Product and Plant Resources.* 3 (4): 38-50. https://www.researchgate.net/publication/263655828.
